# An overview of computational methods for gene prediction in eukaryotes: strengths, limitations, and future directions

**DOI:** 10.1093/bioadv/vbaf222

**Published:** 2025-10-01

**Authors:** Abigail Djossou, Wend Yam D D Ouedraogo, Aida Ouangraoua

**Affiliations:** Department of Computer Science, Universite de Sherbrooke, Sherbrooke, Quebec, J1K 2R1, Canada; Department of Computer Science, Universite de Sherbrooke, Sherbrooke, Quebec, J1K 2R1, Canada; Department of Computer Science, Universite de Sherbrooke, Sherbrooke, Quebec, J1K 2R1, Canada

## Abstract

**Summary:**

Advances in Next-Generation Sequencing (NGS) and machine-learning methods have improved eukaryotic gene prediction. Despite this progress, computational prediction remains crucial for complementing empirical data and annotating newly sequenced genomes, given the complexity of eukaryotic gene structures. Recent deep-learning approaches further enhance accuracy by learning gene-structure patterns directly from genomic sequences, enabling stronger cross-species generalization without predefined gene models. This review introduces a new classification of gene prediction methods—gene-model-based, gene-model-free, and hybrid—and examines representative tools with respect to their algorithmic strategies, input data, strengths, and limitations. It also updates previously reported challenges and outlines new issues arising from modern deep-learning techniques. To support these discussions, we extended the G3PO benchmark of gene-model-based predictors (Augustus, GenScan, GeneID, GlimmerHMM, and SNAP) to additionally include a gene-model-free method, sensor-NN, and a hybrid method, Helixer.

**Availability and implementation:**

Benchmark DNA and protein sequences are available in the G3PO repository (http://git.lbgi.fr/scalzitti/Benchmark_study). Scripts for Augustus and Helixer, along with all prediction outputs, are accessible at https://github.com/UdeS-CoBIUS/GenePredictionReviewBenchmark.

## 1 Introduction

The increasing availability of DNA sequences from various organisms has made computational gene prediction essential for genome annotation in large datasets. Predicting genes in eukaryotes is particularly challenging due to the complexity of their gene structures. Eukaryotes have long intergenic regions that do not code for genes. Moreover, within genes, sequences are divided into expressed regions (exons) and spliced regions (introns), which can be long and complex, making it difficult to identify exon-intron boundaries. Some genomes also contain nested or overlapping genes, which further complicate gene prediction. Identifying all alternative transcripts produced by genes and distinguishing between functional and non-functional ones is also a significant challenge.

Many techniques have been developed to tackle these challenges, including the integration of experimental approaches such as RNA sequencing (RNA-seq) and ribosome profiling (ribo-seq), which have significantly improved the annotation of gene structures and the prediction of protein-coding genes. However, these techniques have limitations that require complementation with computational gene prediction. These limitations include, for instance, the inability to predict unexpressed and lowly expressed genes, the incomplete prediction of gene structures, and the difficulty of integrating different data types. Computational gene prediction remains essential and challenging for comprehensive genome-wide analysis, including the annotation of novel genomes where no empirical sequencing data are available, or the prediction of genes in large datasets from high-throughput genomics projects. Even with empirical data, combining it with computational prediction is essential to achieve the most accurate and complete gene prediction. Computational gene prediction is also crucial for transcriptome analysis, including the identification of alternatively spliced transcripts and expression patterns. While RNA-seq provides data on alternative splicing, computational prediction is important for identifying all possible splice variants.

While computational gene prediction has expanded to include various genetic elements—such as non-coding RNA genes (e.g. miRNAs, lncRNAs, circRNAs) and regulatory regions like promoters and enhancers—most existing tools still focus primarily on protein-coding genes. This is largely due to their well-established role in genome annotation projects and the availability of robust experimental evidence for validation (Wang *et al.* 2004). Nevertheless, there is a growing literature dedicated to the prediction of non-coding RNAs and regulatory features, supported by recent advances in RNA sequencing, machine learning and large DNA language models that enable the simultaneous prediction of multiple feature types (Kim and Lee 2023, Khalid *et al.* 2023, Tang and Ji 2023, Karollus *et al.* 2024, Sanabria *et al.* 2024).

Several methods are primarily designed to accurately recognize the features of protein-coding genes because of their well-established importance in cellular functions and disease mechanisms. These methods use various algorithms and gene structure models. However, achieving both robustness and accuracy remains elusive. Factors such as undetermined nucleotides in genomic sequences, exceptionally short or long introns, small exons, limited intergenic regions, DNA sequencing errors, non-canonical splice sites, overlapping genes, and incomplete gene models can result in incomplete or inaccurate predictions (Meyer *et al* 2020, Mathé and Dunand 2021).

Over the past two decades, computational gene prediction has been the subject of numerous reviews (Claverie 1997, Mathé *et al.* 2002, Brent and Guigo 2004, Wang *et al.* 2004, Do and Choi 2006, Bandyopadhyay *et al.* 2008, Brent 2008, Sleator 2010, Goel *et al.* 2013, Maji and Garg 2013, Ghorbani and Karimi 2015, Hoff and Stanke 2015, Mabrouk *et al.* 2017, Das *et al.* 2018). Signal processing methods in gene prediction have also been extensively reviewed (Mabrouk *et al.* 2017, Das *et al.* 2018). The most recent general reviews on gene prediction methods date back to 2015 (Ghorbani and Karimi 2015, Hoff and Stanke 2015). Gene prediction continues to be a pivotal challenge in genomics. While traditional methods underpin gene annotation, recent innovations emerge from deep learning, which can operate without predefined gene models. This review provides an overview of protein-coding gene prediction techniques, covering both traditional and contemporary tools. It touches upon both widely recognized and lesser-known methods. For this reason, the descriptions of methods are kept brief. For detailed insights into specific techniques, readers are referred to the more specific literature indicated in this review. The purpose of this review is to familiarize developers with foundational gene prediction techniques and current methodological challenges, and to provide a valuable resource for both developers and users within the community.

We start by summarizing the recent improvements and persistent challenges of computational gene prediction. We then describe the various types of information used in computational gene prediction. Next, we categorize gene prediction methods into three main groups, describing the algorithms and tools within each. We then discuss the case of combiners and pipelines, which are two categories of tools developed to improve the accuracy and completeness of predicted gene models by integrating results from multiple gene prediction tools. We also discuss major annotation providers. Finally, we present tools and benchmarks for evaluating these methods, along with an experimental comparison of a subset of gene prediction tools. Our discussion concludes with an examination of the challenges faced by gene prediction tools, including well-documented issues that remain unresolved and new challenges introduced by gene model-free approaches.

The key insights from this manuscript are:

- An updated overview of computational gene prediction methods in eukaryotes, including modern gene model-free and hybrid techniques;
- A comparative benchmark of selected gene prediction tools, providing insights into their performance; and
- An analysis of the strengths and limitations of current approaches, emphasizing the latest understanding of gene expression.

## 2 Recent improvements in gene prediction

In recent years, there have been several advancements in computational gene prediction, including improvements to existing annotation pipelines, the use of experimental techniques such as RNA sequencing and ribosome profiling, and the integration of deep learning models to enhance accuracy. With the substantial increase in available RNA-seq and protein homology data, several tools—such as TSEBRA (Gabriel *et al.* 2021), BRAKER3 (Gabriel *et al.* 2024), and GeneMark-ETP (Brůna *et al.* 2024)—have evolved to integrate gene predictions with RNA-seq and protein evidence for more precise gene annotation. Some pipelines, such as BRAKER3 (Gabriel *et al.* 2024) and MAKER (Holt and Yandell 2011), even support the use of long-read RNA-seq data to improve gene structure annotation, especially for complex gene architectures that short reads often fail to resolve.

Many prediction tools now integrate deep learning models, such as convolutional neural networks and recurrent neural networks, to predict gene structures with higher accuracy. For example, GeneMark-EP+ (Brůna *et al.* 2020) and HELIXER (Holst *et al.* 2023) can incorporate evidence derived from deep learning methods. Other tools, such as AUGUSTUS (Stanke and Waack 2003) and GeneMark (Borodovsky and McIninch 1993), can also be used in combination with deep learning tools through BRAKER2 (Brůna *et al.* 2021), which incorporates hints from deep learning-based predictions.

Transfer learning and cross-species prediction have also advanced the field of gene prediction. These approaches enable the use of models trained on well-annotated model organisms to predict genes in non-model organisms. For instance, HELIXER (Holst *et al.* 2023), trained on Arabidopsis data, demonstrates generalization to other species through fine-tuning or retraining with smaller datasets. Other tools, such as GeneMark-ES (Lomsadze *et al.* 2005), AUGUSTUS (Stanke and Waack 2003), and BRAKER (Brůna *et al.* 2021), have incorporated comparative models or evidence from related species to enhance performance.

Advancements in ribosome profiling (Ribo-seq) techniques have significantly enhanced the annotation of small open reading frames (sORFs) (Mudge *et al.* 2022), a relatively new class of small genes that have traditionally been overlooked in gene annotation due to the complexity of identifying very short sequences. Ribo-seq provides detailed insights into translation processes and offers direct evidence of sORF translation (Dong *et al.* 2023, Simoens *et al.* 2023). Complementing these experimental techniques, various computational tools—such as sORF Finder (Hanada *et al.* 2010, Banerjee *et al.* 2021), CPPred-sORF (Tong *et al.* 2020), and MipepID (Zhu and Gribskov 2019)—predict the coding potential of sORFs using diverse approaches, ranging from codon substitution analysis to machine learning. For a recent survey on experimental and computational methods for the identification of small proteins, see Beals *et al.* (2024).

In addition to the established methods described above, there is a growing interest in applying large language model (LLM) concepts to genomic sequences—so-called DNA language models. The principle is straightforward: treat DNA as a language and learn its “grammar” by identifying statistical patterns and long-range relationships between k-mers, in the same way that language models capture words and syntax. Once trained, these models can be adapted to a wide variety of tasks, from predicting protein-coding genes to identifying regulatory regions such as promoters and enhancers. An example is **GROVER** (Sanabria *et al.* 2024), a foundation model trained on large genomic datasets that can be fine-tuned for almost any genomic prediction problem given the right data. Similar approaches include **DNABERT** (Ji *et al.* 2021), which adapts the BERT architecture to nucleotide sequences, and **Nucleotide Transformer** (Lopez *et al.* 2023), a large-scale model trained on hundreds of species. Unlike traditional tools designed for a single purpose, these models are inherently flexible and can predict multiple types of genomic features within the same framework. This flexibility suggests that they may soon become an integral part of gene annotation pipelines, complementing—and in some cases replacing—more specialized approaches.

## 3 Persistent challenges of computational gene prediction for eukaryotes

The goal of gene identification in a genomic sequence is to pinpoint gene regions, define their boundaries, and determine their internal structure. Eukaryotic genomes are more complex than prokaryotic genomes, containing large intergenic DNA regions. Many eukaryotic genes consist of multiple exons separated by large introns (Pevzner 2000). Genes can also overlap on the same or opposite strands. This structural complexity demands not only the identification of gene boundaries but also the configuration of their exon–intron structures, which complicates gene prediction.

Identifying single-exon genes also presents a significant challenge in gene prediction. Recent studies, such as that by Wegrzyn *et al.* (Leaves Project), indicate that many single-exon genes may not be genuine. Only a small, conserved subset seems to be functional, particularly in plants. The suggestion to disregard mono-exonic genes lacking a protein domain underscores the importance of thoroughly evaluating these genes.

Alternative splicing, which allows a single gene locus to produce multiple transcript variants and protein products, complicates gene prediction. Although alternative splicing is common in eukaryotes (Matlin *et al.* 2005), many existing methods predict just one isoform per gene, overlooking this diversity.

Small open reading frames (sORFs), typically defined as coding sequences shorter than 100 amino acids, have recently emerged as an important source of functional microproteins in eukaryotic genomes (Andrews and Rothnagel 2014, Couso and Patraquim 2017). Many sORFs were historically overlooked in gene annotation due to size-based filtering thresholds and the difficulty of detecting their products experimentally. Advances in ribosome profiling (Ribo-seq) have greatly facilitated the identification of actively translated sORFs by providing nucleotide-resolution maps of ribosome occupancy (Ingolia *et al.* 2011). Complementing this, mass spectrometry-based proteomics provides direct evidence for the existence of microproteins, making it one of the most reliable large-scale approaches for confirming protein-coding genes (Slavoff *et al.* 2013, Ma *et al.* 2016). This technique has been used for years to support genome annotation by validating translation events and refining gene models.

The integration of sORF discovery into multi-omics frameworks is gaining momentum, combining transcriptomics, translatomics, and proteomics to improve annotation accuracy. Several computational tools and pipelines already implement this approach. For example, ORFquant (Bazzini *et al.* 2014) combines Ribo-seq and RNA-seq to detect and quantify ORFs; RiboTaper (Calviello *et al.* 2016) uses Ribo-seq periodicity to identify translated regions; and PROTEOFORMER (Verbruggen *et al.* 2019) integrates RNA-seq, Ribo-seq, and mass spectrometry to provide experimental evidence for annotated and novel proteins. These integrative strategies are particularly promising for uncovering overlooked coding sequences, including those embedded in non-coding RNAs or alternative reading frames.

Computational gene prediction also faces limitations related to training data. Many eukaryotic genomes lack comprehensive, high-quality annotations necessary for training prediction models. As a result, existing methods are often biased toward well-studied model organisms such as humans and Arabidopsis, leading to poor generalizability across species. Additionally, available training data typically lack tissue-specific expression information, which can result in the omission of genes expressed only under specific conditions.

Another challenge relates to the use of predefined models, such as HMMs, to represent gene structure. Several methods rely on a predefined gene structure model from the outset, which makes them less adaptable to unusual genome structures or novel organisms.

These challenges call for continuous improvements in gene prediction algorithms that incorporate multi-omics data for gene annotation in eukaryotes.

## 4 Information used for gene prediction

In gene prediction tool development, the choice of information and the algorithm used to process it are crucial. Gene structures are typically predicted using four types of information: signal sensors, content sensors, gene similarity, and experimental data (Stormo 2000). The first two are considered intrinsic information, while the latter two are classified as extrinsic information.

*Intrinsic information*, also known as ab initio information, is derived directly from the genomic sequence. Methods that rely on intrinsic information identify gene functional sites using features such as signal sensors within the sequence and content statistics (Mathé *et al.* 2002). Signal sensors, which are essential for gene expression regulation, include specific sequence patterns such as splice sites and start and stop codons (Wang *et al.* 2004). Content statistics help differentiate coding from non-coding regions in a genomic sequence. Gene prediction is further improved by considering factors such as species-specific codon biases, GC content, and nucleotide composition (Goel *et al.* 2013). Hidden Markov models are widely used in computational gene prediction based on this type of information.

*Extrinsic information* plays a crucial role in various gene prediction approaches, including homology-based, comparative genomics-based, and sequencing-based techniques. Homology-based methods identify genes in a target genome by detecting similarities with known genes or proteins from other close organisms (Mathé *et al.* 2002, Keilwagen *et al.* 2016). Comparative genomics approaches infer gene structures by aligning multiple genomes to identify conserved coding regions across species (Birney *et al.* 2004, Frazer *et al.* 2004). Additionally, sequencing technologies such as RNA-seq and Ribo-seq provide critical extrinsic data by revealing active transcription and translation processes, which further helps in accurately annotating genomes (Ingolia *et al.* 2009, Wang *et al.* 2009a,b). Other sequencing methods like ChIP-seq, ATAC-seq, and Hi-C-seq can also provide extrinsic information by mapping protein–DNA interactions, chromatin accessibility, and 3D genome architecture (Johnson *et al.* 2007, Lieberman-Aiden *et al.* 2009, Buenrostro *et al.* 2013).

The use of extrinsic information, such as gene homology, RNA-seq and comparative genomics, has become increasingly important in improving the accuracy of computational gene prediction. In particular, RNA-seq provides critical information on gene expression and transcript diversity, enabling more precise mapping of exons and introns, particularly in complex eukaryotic genomes (Conesa *et al.* 2016). While the integration of such data significantly improves gene prediction, it also presents challenges, including variability in data quality and the need for robust algorithms (Kellis *et al.* 2014). As genomic technologies advance, the effective use of empirical data, combined with machine learning, will be key to refining gene prediction tools and updating gene models to reflect new biological knowledge (Kellis *et al.* 2014).

## 5 Three main categories of gene prediction methods

**Figure 1.**
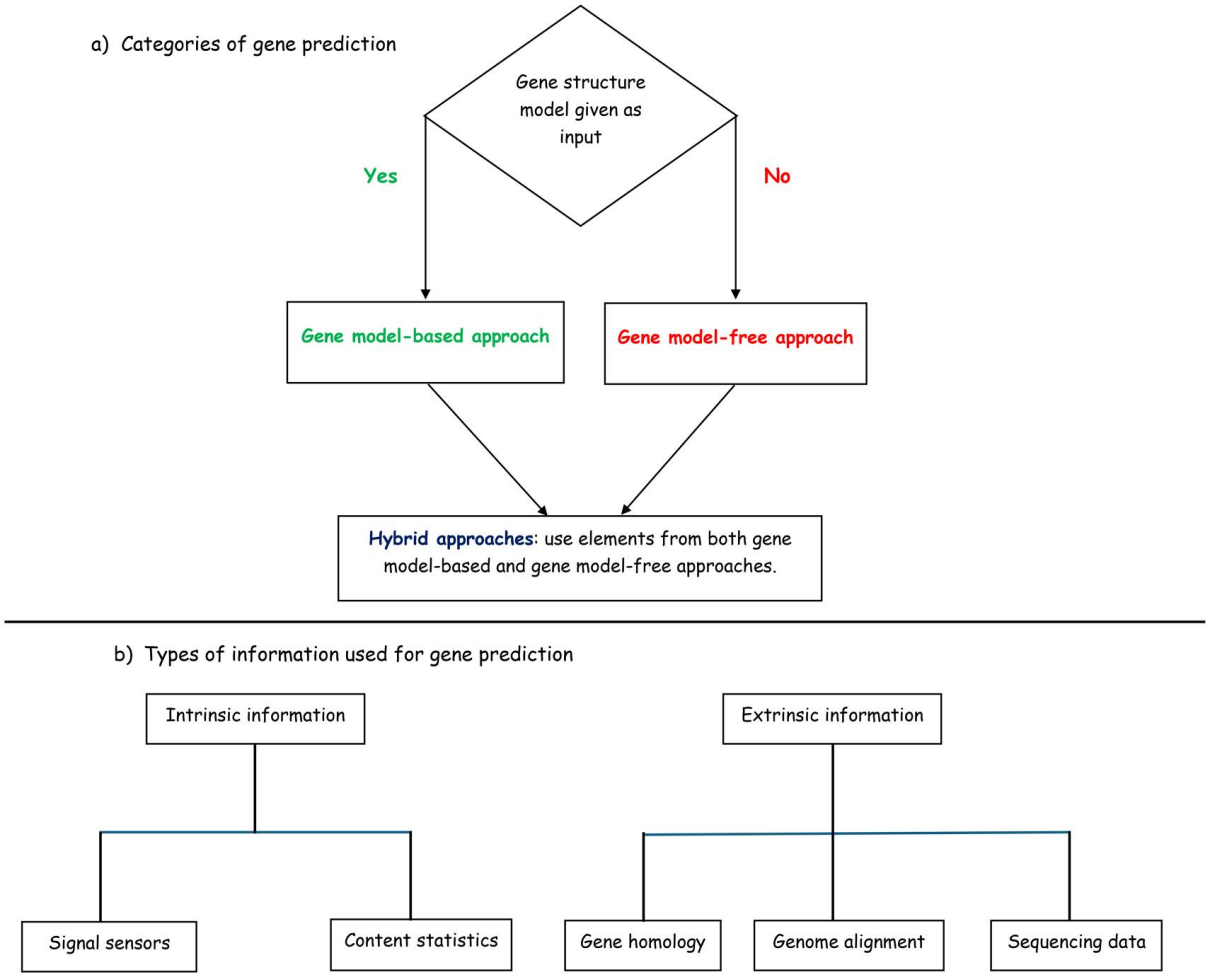
Categories of gene prediction methods (a) and types of information used by each method (b).

Over time, numerous algorithms have emerged to predict gene structures. In this review, we propose a novel classification for gene prediction methods: gene model-based, gene model-free, and hybrid approaches. This classification extends beyond traditional ones, focusing on the technique rather than the type of information used (see Fig. 1). This framework is presented as a practical, non-exhaustive guide to help readers navigate the increasing diversity of methods in the field, rather than as a definitive or universally adopted taxonomy. We acknowledge that overlaps exist between categories and that the landscape of gene prediction evolves rapidly; a formal classification would ideally be developed through broader community consensus, such as a consortium effort. Nevertheless, this structure offers a useful conceptual overview, particularly for readers new to the field, and complements the broader survey of tools provided in this review.

**Gene model-based approaches** use known patterns or structures of genes to identify gene locations. They use either features within the DNA sequence or reference sequences from other species. Essentially, they leverage existing knowledge about gene models. Examples include methods that use hidden Markov models (HMMs). Hence, the methods in this category are based on a predefined description of the structural components and organization of a gene, called a gene model. In gene model-based approaches, gene prediction is performed against predefined gene models. These gene models provide information about the organization of the various elements that form a gene, including exons, introns, promoters and regulatory regions, untranslated regions, splicing, transcription, and translation start and stop sites. Gene models are constructed based on experimental evidence and are explicitly provided as input to model-based approaches.

**Gene model-free approaches** directly learn from the data, extracting insights without conforming to predefined gene model patterns. Gene model-free approaches are designated as such because they do not start with a predefined gene model. Instead, they learn patterns of a gene model directly from training data. This learning is achieved through algorithms that can adaptively infer structures and relationships within the training data, without prior knowledge. Their flexibility allows them to detect patterns that might be overlooked by gene model-based methods. Examples of these techniques are artificial neural networks (ANNs) and decision trees.

**Hybrid approaches** represent an evolving category of methods, combining elements of both gene model-based and gene model-free approaches. They use predefined gene models alongside data-driven techniques to improve gene prediction accuracy.

This tripartite classification aims to provide a more comprehensive perspective on gene prediction methods, reflecting recent advancements in the field. However, it is important to note that, in machine learning, the term model is also used to describe the representation of a process learned by a machine learning algorithm. Thus, in this context, a model is the result of the learning algorithm itself. For example, an Artificial Neural Network (ANN) is a model in the context of machine learning, but it can be considered a gene model-free approach in our classification if its learning algorithm does not take as input a predefined gene model.

In the subsequent sections, we discuss some representative methods in the three categories: gene model-based, gene model-free, and hybrid approaches. While we discuss only a few methods, a comprehensive list is available in the Supplementary Materials’ Tables, available as supplementary data at *Bioinformatics Advances* online. Table 1, available as supplementary data at *Bioinformatics Advances* online lists gene model-based tools, Table 2, available as supplementary data at *Bioinformatics Advances* online features gene model-free tools, Table 3, available as supplementary data at *Bioinformatics Advances* online describes hybrid tools, and Table 4, available as supplementary data at *Bioinformatics Advances* online lists combiners and pipelines.

For readers keen on the historical context of these tools, Table 5, available as supplementary data at *Bioinformatics Advances* online in the supplementary materials indicates the original organisms for which the tools were designed. This can shed light on a tool’s initial purpose and potential biases. However, many tools, especially those marked with an asterisk (*), have often been retrained on other organisms. Thus, consulting the latest documentation or information on a tool is crucial before selection. Lastly, Table 6, available as supplementary data at *Bioinformatics Advances* online helps users in selecting a tool based on features like web server availability, source code access, or standalone software provision.

## 6 Gene model-based approaches for computational gene prediction

Gene model-based approaches for gene prediction rely on complete and explicit gene structure models as input to analyze genomic sequences. These methods are built on biological models that describe gene architecture, incorporating detailed knowledge of genomic features such as exon and intron sequences, splice sites, and promoter regions.

In these methods, gene prediction is performed by comparing sequences of interest with predefined models to identify matches that suggest the presence of gene structures. The process can use both intrinsic information derived directly from the sequence and extrinsic information, such as sequence alignment and conservation across species, to support gene prediction and validate identified genes.

The primary characteristic of model-based approaches is their reliance on predefined references, enabling prediction through alignment with established gene models. However, because of this dependence on known models or reference gene sequences, these approaches face limitations in detecting novel or atypical gene structures, particularly in genomes that are poorly studied or exhibit high genetic diversity. Because of these limitations, gene model-based methods are usually used as parts of gene prediction pipelines.

As shown in Table 1, available as supplementary data at *Bioinformatics Advances* online, a comprehensive list of gene model-based tools is provided in the Supplementary Materials, available as supplementary data at *Bioinformatics Advances* online.

### 6.1 Hidden Markov models (HMMs)

Hidden Markov Models (HMMs) represent probability distributions over sequences of observations using a Markov process with hidden states. Essentially, HMMs are statistical models that decode information from hidden symbols. A key aspect of HMMs in gene prediction is their reliance on Dynamic Programming (DP) for efficient computation. This allows HMMs to handle the complexities of eukaryotic gene structures, making them a preferred choice for many gene prediction tools. They have become popular in gene prediction for three main reasons:

- Their models have an intuitive analogy with gene structures;
- They have a strong mathematical basis that supports detailed analysis;
- Over time, many gene prediction algorithms have been developed based on HMMs (Stormo 2000).

Generalized HMMs (GHMMs) are among the advances in HMMs that have shown remarkable precision in gene prediction. Historically, HMMs were foundational in gene prediction, leading to the creation of many tools. Several ab initio gene prediction tools use HMMs to detect distinct gene features such as UTRs, exons, and introns (Techa-Angkoon *et al.* 2019). When processing an input sequence, HMMs can pinpoint the most probable structural features, providing a comprehensive overview of gene structure. It should be noted that the extension of HMM-based methods with homology-based approaches, which leverage evolutionary conservation and species similarity, often achieves greater accuracy than HMMs.

**GenScan** (Burge and Karlin 1997) is an ab initio gene prediction tool based on a probabilistic model of human gene structure. It detects gene signals and accounts for the C + G content density. While it can predict multiple genes in a sequence, it does not address overlapping segments or alternative splicing. Its successor, **GenomeScan** (Yeh *et al.* 2001), enhances prediction by incorporating protein similarity. **Twinscan** extends the GenScan model by leveraging the homology between two genomes and adding a conservation parameter (Korf *et al.* 2001, Flicek *et al.* 2003).

**GeneScout** uses multiple HMM models for predicting functional sites and another HMM model for coding potentials (Yin and Wang 2004). It uses a directed acyclic graph to identify coding regions, optimized with dynamic programming.

**AUGUSTUS** (Stanke and Waack 2003, Stanke *et al.* 2006a,b; Keller *et al.* 2011, König *et al.* 2016) has evolved significantly over time. Originally created to identify protein-coding genes in eukaryotic genomes using a Generalized Hidden Markov Model (GHMM), its capabilities have expanded. It now allows the use of either a GHMM or a Conditional Random Field (CRF) based on species-specific parameters to enhance prediction precision. AUGUSTUS integrates external evidence, predicts multiple transcripts for a single genomic sequence, and has added a protein model for more refined predictions. One of its latest features supports simultaneous gene prediction across various genomes, highlighting its adaptability in gene prediction.

**Genie** (Reese *et al.* 2000) uses a GHMM to model genes in DNA, optimizing probabilities with machine learning and using neural networks for splice site prediction.

**SNAP** (Korf 2004) adjusts to different organisms, underscoring the importance of species-specific gene prediction tools, given the sensitivity of such predictions to specific species parameters.

**GeneWise** (Birney *et al.* 2004) predicts gene structures by aligning a protein sequence to genomic DNA, drawing on principles from hidden Markov models.

Other tools include **GeneMark** (Borodovsky and McIninch 1993), based on a Bayesian algorithm; **HMMgene** (Krogh 1997), focused on prediction accuracy; **SLAM** (Cawley and Pachter 2003), which aligns two genomic sequences to predict splice variants; and **GAZE** (Howe *et al.* 2002), which integrates multiple sources of evidence such as ab initio predictions, homology alignments, and RNA data into a unified gene model.

### 6.2 Dynamic programming (DP)

Dynamic Programming (DP) is a fundamental algorithmic approach that finds extensive application across various computational biology methods, including those used in gene prediction. Its ability to break down complex problems into simpler sub-problems makes it particularly effective in dealing with the complexity of gene structures. DP forms the computational backbone for numerous methods. Its implementation in HMMs is a prime example of its versatility and power. This section focuses on the broader applications of DP in gene prediction. Several gene prediction tools use this technique:

**GeMoMa** (Gene Model Mapper) (Keilwagen *et al.* 2016, Keilwagen *et al.* 2019) is designed for homology-based gene prediction. It uses RNA-seq data to enhance prediction accuracy. GeMoMa predicts genes in a target genome using annotated genes from a related reference genome, refining predictions with intron position conservation and RNA-seq data. This makes it valuable for predicting genes in non-model organisms with limited annotated gene sets.

**GeneBuilder** (Milanesi *et al.* 1999) emerges as a tool that predicts coding regions and functional signals by probing different methodologies and finding alignments in protein and EST databases. Its dynamic programming application aids in developing potential genetic structure models, thus providing an avenue to consider alternative gene model versions based on protein homology and model optimization using variable parameters.

**Fgene** (Salamov and Solovyev 2000) has the capability to predict internal exons, as well as 5′ and 3′ exons, by evaluating contextual features through linear discriminant functions. The tool harnesses dynamic programming techniques to ascertain optimal exon combinations, facilitating the formulation of gene models.

**GREAT** (Genome Recognition and Exon Assembly Tool) (Gelfand *et al.* 1996a,b) integrates codon usage and splicing signal nucleotide frequencies into its gene structure prediction scoring function. This tool, grounded in dynamic programming, merges multiple exon quality indicators using dynamic vector programming.

**GlimmerM** uses a dynamic programming algorithm to assess potential exon combinations for a gene model, selecting the best combination using interpolated Markov models (IMMs) (Salzberg *et al.* 1999).

Other tools, such as **GeneID** (Guigó *et al.* 1992), which uses a probabilistic graph model inspired by GHMMs, and **GAP III** (Gene Assembly Program III) (Xu *et al.* 1994a,b), which reconstructs gene models from predicted exons, also leverage dynamic programming. DP significantly improves the gene prediction capabilities of these tools.

### 6.3 CRF-based methods

Building upon the foundational role of HMMs in gene prediction, CRF-based methods (Conditional Random Fields) have emerged as a significant shift in the approach to gene prediction. Unlike HMMs, which are generative models focusing on the probability distributions of sequences, CRFs are discriminative models. They rely on the differentiation between various classes of sequences, offering a more direct approach to sequence classification.

This shift from generative to discriminative modeling toward more flexible machine learning approaches is exemplified by tools like **Conrad** (DeCaprio *et al.* 2007) and **CRAIG** (Bernal *et al.* 2007). Compared to HMMs, CRFs directly model the conditional probability of hidden states given observations, while HMMs model the joint probability of hidden states and observations. The inference algorithms used in methods based on CRFs are essentially the same as those used in HMMs (Ng and Jordan 2001).

This discriminative approach has been particularly effective in the context of the wealth of data provided by RNA-seq technologies, which have enabled a more empirically driven understanding of gene structures, including alternative splicing events. While CRFs improved upon HMMs, they have now been superseded by deep learning models, which allow for learning hierarchical features and long-range dependencies directly from raw sequences.

## 7 Gene model-free approaches for computational gene prediction

Gene model-free approaches for gene prediction are driven by data, relying on information within genomic sequences without the need for predefined gene models or reference gene structures. They consider gene annotation as a base pair-wise classification problem. These methods analyze intrinsic features and indicators in the genomic data, such as sequence motifs and splicing signals, to identify potential gene structures. Machine learning techniques are applied to establish relationships from large training datasets, enabling predictions about gene presence and structure through inductive learning.

By scanning sequences for similarities, conserved variations, or other biologically relevant signals, these approaches continuously adjust and refine their prediction models as new data become available. This adaptability makes them particularly effective at identifying previously unrecognized gene structures, including those within poorly characterized or highly variable sequences. However, the effectiveness of gene model-free approaches relies heavily on the availability and quality of training data. Comprehensive and representative datasets are essential, as insufficient or biased data can substantially reduce prediction accuracy.

Moreover, most gene model-free methods produce only base-pair classifications, not complete gene models. Therefore, they are typically used in hybrid approaches to generate comprehensive gene models. As shown in Table 2, available as supplementary data at *Bioinformatics Advances* online, a comprehensive list of gene model-free tools is provided in the Supplementary Materials, available as supplementary data at *Bioinformatics Advances* online.

### 7.1 Decision trees

A decision tree is a flowchart-like structure that illustrates potential outcomes of a series of decisions. Starting from a single node, it branches out into various outcomes, with each leading to further possibilities, forming a tree-like diagram. Decision trees effectively learn from large datasets and are known for their accuracy. They have provided valuable insights in biology (Clare and King 2003) and are widely used in operations research and decision analysis (Breiman *et al.* 1984). Salzberg (1995) demonstrated the capability of decision trees to accurately differentiate coding from non-coding DNA, even in short sequences. Since then, gene recognition has been viewed as a parsing problem, a probabilistic reasoning task, and a classification challenge (Salzberg *et al.* 1998).**genBlastDT** (She *et al.* 2010) is a homology-based gene prediction tool. Like GeneWise, it processes a query protein sequence and a target DNA sequence. Its distinct feature is the use of decision trees to detect intron regions.

### 7.2 Artificial neural networks

Artificial Neural Networks (ANNs) are machine learning models inspired by biological neural networks, comprising interconnected neurons (Wu and McLarty 2012). Their architecture typically includes one hidden neuron layer between the input and output layers. They excel in solving moderately complex problems. Deep neural networks (DNNs) extend ANNs with multiple layers between input and output, allowing them to model more complex and hierarchical data patterns such as gene locations and structures in genomes (Goel *et al.* 2013).

Deep learning has significantly impacted various scientific fields, including genomics. In particular, for sequential data such as DNA sequences, recurrent neural networks (RNNs) allow maintaining a memory of past inputs through internal loops. They have been used successfully for gene annotation in eukaryotes (Stiehler *et al.*2021) and prokaryotes (Amin *et al.* 2018). Recent advancements in neural network architectures have introduced models with unique features beneficial for gene prediction, including attention mechanisms and Transformer models (Vaswani *et al.* 2017). Attention mechanisms enhance a model’s sequence processing by allowing it to focus selectively on specific input segments. In gene prediction, this means prioritizing certain genome regions that are more informative. Building upon attention mechanisms, Transformer models efficiently handle large datasets. Their self-attention capability is crucial for understanding complex relationships within genomic sequences. While these recent advancements in neural network architectures are still emerging in genomics, they hold promising potential for future developments in gene prediction.

**GIN** (Gene Identification using Neural nets and homology information) is a pioneering tool in this category, which combines homology data from diverse protein databases (Goel *et al.* 2013). Designed to minimize false positives, GIN uses a backpropagation neural network. It can predict multiple genes in genomic DNA and is more accurate than methods like GeneID and GeneParser when using homology data. However, its accuracy diminishes without homology information.

The original version of **GRAIL** (Gene Recognition and Analysis Internet Link) (Uberbacher and Mural 1991) uses a neural network-based analysis with a fixed-length sliding window approach to evaluate the coding potential of regions within a DNA sequence. The method relies on the computation of protein-coding sensor signals for a 99-base sequence window, which are then evaluated by a neural network. GRAIL1 was later extended to GRAIL2, which also integrates gene model elements—including splice sites, translation start sites, and domain sensor signals—to improve the method’s accuracy.

The initial version of **Helixer** (Stiehler *et al.* 2021) uses DNNs for ab initio cross-species gene annotation. It uses a special kind of RNN, a bidirectional long-short term memory (BLSTM) network, which allows the processing of sequential inputs starting from both ends (Hochreiter and Schmidhuber 1997). Its model is trained to distinguish between four types of regions: intergenic, untranslated (UTR), coding (CDS), and introns. Stiehler *et al.* (2021) showed that Helixer made substantial performance gains compared to AUGUSTUS and also exceeded the quality of some references in consistency with independent RNA-seq data. Recently, Helixer was extended by combining it with an HMM-based post-processing step to enable the production of finalized gene models (Holst *et al.* 2023). In this review, we refer to the initial version as HelixerInit, while the most recent hybrid version is called Helixer.

More recently, **sensor-NN**, an advanced ab initio gene prediction tool, was developed (Baker *et al.* 2023). It uses a neural network to analyze DNA sequences and predict coding regions. It relies on algorithmic sensors that generate features from nucleotide windows, which are then used as inputs to the neural network. The sensors measure various aspects of the sequence, such as codon periodicity, k-mer frequencies, GC content, and proximity to start and stop codons, providing a rich feature matrix for each analyzed segment. The neural network is trained to distinguish between coding and non-coding regions, offering precise predictions even in the absence of comparative sequencing data or reference annotations. Sensor-NN was compared to well-established gene prediction tools on the G3PO dataset (Scalzitti *et al.* 2020), where it demonstrated superior ability to generalize across diverse non-model species.

## 8 Hybrid approaches for computational gene prediction

Hybrid approaches integrate the strengths of both gene model-based and model-free approaches, combining algorithms from both to benefit from the accuracy of statistical models and the flexibility of data-driven techniques. This usually improves gene prediction accuracy, particularly when dealing with complex or unconventional genomic contexts. Hybrid methods use the precision of gene model-based techniques for known gene structures and the adaptability of gene model-free techniques for discovering new genes. This cohesive strategy offers a comprehensive solution to gene prediction, capable of tackling the varied challenges presented by different genomic scenarios. As advancements in machine learning and statistical modeling continue, hybrid methods are able to provide a richer and more comprehensive understanding of gene structures across diverse genomes. As shown in Table 3, available as supplementary data at *Bioinformatics Advances* online, a comprehensive list of hybrid tools is provided in the Supplementary Materials, available as supplementary data at *Bioinformatics Advances* online.

**Helixer** (Holst *et al.* 2023) combines deep learning (HelixerInit) with a Hidden Markov Model (HMM) for post-processing the results of HelixerInit. Unlike many traditional tools that typically predict a single transcript per gene, Helixer captures the complexity of eukaryotic genes, which can produce multiple transcripts via alternative splicing. Additionally, Helixer’s web-based interface eliminates software installation needs, marking a leap in applying deep learning to gene prediction.

**Tiberius** (Gabriel *et al.* 2024) integrates convolutional neural networks (CNNs) and bidirectional long short-term memory (biLSTM) layers with a differentiable HMM layer to predict gene structures from genomic sequences. Unlike Helixer, which uses a separate gene model-based post-processing step, Tiberius incorporates HMMs directly into both the training and inference processes, allowing the model to account for gene structure during optimization.

**GenViewer** (Milanesi *et al.* 1993) is designed for the recognition of gene coding regions in human nucleotide sequences. It uses a combined technique that involves searching for potential splice sites, creating a set of potential coding regions, and estimating their coding potential. The tool operates in two modes: automated and dialog. The automated version, ORFJ, follows a linear recognition scenario using standard programs, while an advanced interface allows for in-depth analysis and interactive prediction of the results.

**GRAIL2** (Gene Recognition and Analysis Internet Link) (Shah and Uberbacher 1994) builds upon GRAIL1 (Uberbacher and Mural 1991) by combining neural network-based analysis with model elements such as splice site recognition. It identifies genes in DNA by pinpointing protein-coding region features and their boundaries (Murthy and Varma 2014). A multilayer feed-forward neural network evaluates DNA sequences within sliding windows. GRAIL2 improved on the capacity of GRAIL1 to identify short exons (Xu *et al.* 1994a,b) by considering variable-length windows. Another update in 1996 used a multi-agent neural network system for better coding region identification (Xu *et al.* 1996). The latest version, GrailExp, integrates homology information (Xu *et al.* 1996).

**GeneParser** analyzes genomic DNA sequences for protein gene structure (Snyder and Stormo 1995). It builds on GRAIL’s methodology with an added recursive optimization procedure through dynamic programming. This program predicts exons and introns using content and site statistics combined with neural networks and database searches. Reports suggest its accuracy surpasses GRAIL and GeneID (Bishop 1998).**mGene** (Schweikert *et al.* 2009) is a gene-prediction tool tailored for eukaryotic genomes. It integrates generalized hidden Markov models (gHMMs) with the precision of Support Vector Machines (SVMs). This combination allows mGene to model gene structures accurately and offers a significant advantage in predicting gene functions, especially in nematode genomes.

**MORGAN** (Salzberg *et al.*1998) aims to identify genes in vertebrate DNA sequences. While it uses methods like dynamic programming and Markov models, its distinguishing feature is the decision-tree classifier. To differentiate coding DNA from non-coding sections, MORGAN uses the OC1 decision-tree system developed by Murthy *et al.* (1993). It uses sets of decision trees to calculate probabilities through a unique scoring function.

## 9 Combiners and pipelines for gene prediction

Combiners and pipelines are two categories of tools developed to improve the accuracy and completeness of predicted gene models. Combiners allow merging the outputs of multiple gene prediction methods, while pipelines are an automated workflow of tools and steps executed in a predefined order for gene annotation. Table 4, available as supplementary data at *Bioinformatics Advances* online provides a comprehensive list of combiners and pipeline tools.

### 9.1 Combiners

Gene prediction programs use varied techniques and insights on gene features, resulting in diverse predictions for the same sequence. While some programs might typically underperform, they can occasionally offer superior predictions for certain sequence types. Therefore, combining results from multiple programs can enhance gene prediction precision. Numerous tools have been developed to analyze and merge outcomes from different gene prediction applications.

Murakami and Takagi (1998) introduced **GeneScope**, a tool that integrates predictions from four programs: FEXH, GeneParser, GENSCAN, and GRAIL2 in five different ways. Tests on DNA sequences with both partial and complete genes showed that the HIGHEST method, which selects the highest-scoring regions from the programs as exon candidates, improved the approximate correlation (AC) by 3%–5%. The AC for each sequence is calculated as:


$$AC=\frac{1}{2}\;[\frac{TP}{TP+FN}+\frac{TP}{TP+FP}+\frac{TN}{TN+FP}+\frac{TN}{TN+FN}]\;-\;1$$

where TP is the count of coding nucleotides predicted as coding; FN denotes coding nucleotides predicted as noncoding; TN stands for noncoding nucleotides predicted as noncoding; and FP refers to noncoding nucleotides predicted as coding (Murakami and Takagi 1998). GeneScope’s methods can be applied to other prediction programs, with accuracy increasing as more programs are combined.

**GENFOCS** provides a platform for comparing results from gene prediction tools for selected organisms such as *Arabidopsis thaliana*, humans, and rice (Anto *et al.* 2008). It compares various gene search tools by analyzing their predictions on a given DNA sequence and is suitable for both eukaryotes and prokaryotes.

**TSEBRA** (Tool for Selecting the Best RNA-Seq-based gene predictions) integrates multiple sources of gene predictions, focusing on RNA-Seq data, to enhance the overall quality of gene annotations (Gabriel *et al.* 2021). By assessing and choosing the most accurate gene structures, TSEBRA proves invaluable in genome annotation efforts, especially when integrated into frameworks like BRAKER.

**MAKER** functions both as a pipeline and a combiner (Cantarel *et al.* 2008, Holt and Yandell 2011). It annotates new genomes by merging outputs from various gene prediction methods, including ab initio predictors, protein homology data, and expressed sequence tags (ESTs). As a combiner, MAKER produces a consensus gene annotation by leveraging multiple prediction methods.

**GAF** (GeMoMa Annotation File), part of the GeMoMa toolkit, selects the best gene model prediction based on evidence like RNA-seq data (Keilwagen *et al.* 2019). It refines the overall set of gene predictions by evaluating multiple gene models.

**Combiner** integrates multiple sources of evidence for a genomic sequence, including gene prediction locations from ab initio methods, protein sequence alignments, expressed sequence tag and cDNA alignments, and splice site predictions (Allen *et al.* 2004). Its results show that combining outputs from multiple gene prediction tools and other sources of evidence can outperform individual tools.

**EVidenceModeler** (EVM) (Haas *et al.* 2008) is an automated eukaryotic gene structure annotation tool that builds on the methods of Combiner. Paired with the Spliced Alignment Assembly Program (PASA) (Haas *et al.* 2003), it predicts protein-coding genes and alternative spliced isoforms.

**GLEAN** is a versatile tool that has been applied to a variety of genomic projects. Initially developed as part of the honey bee (Apis mellifera) genome project, it combines multiple gene lists to increase the number of gene models by merging prediction sets (Elsik *et al.* 2007). GLEAN uses latent class analysis to create consensus genetic models, maximizing the probability of sites in each model. Beyond its role in the honey bee genome project, GLEAN has also been used for genomic studies in several other species, including the red flour beetle (Park *et al.* 2008), bovine cattle, Toxoplasma gondii, and Plasmodium vivax.

**Evigan** (EValuated Integrated Gene ANnotation) integrates multiple gene prediction sources using a hidden semi-Markov model (HSMM) (Liu *et al.* 2008). It combines predictions from several tools such as AUGUSTUS, GeneMark, GENSCAN, and SNAP, together with extrinsic information such as BLAST hits, splice site prediction, protein homology, and mRNA alignments.

### 9.2 Pipelines

In the context of gene prediction, pipelines are automated workflows designed to streamline the process, from raw genome sequence input to the final annotated genome output. They integrate multiple tools and methods, reducing manual intervention and ensuring reproducibility. The primary advantage of pipelines is their ability to provide a comprehensive and consistent approach to gene prediction.

**GALBA** (Brůna *et al.* 2023a,b) is an advanced pipeline that incorporates three main components: miniprot for splice-aligning protein sequences to genomes, miniprothint for scoring these alignments, and AUGUSTUS for gene prediction. After an initial prediction, genes with full evidence support undergo further training, enhancing the accuracy of the final gene set. Built on the BRAKER codebase in Perl, GALBA is tailored for use with closely related species’ reference proteomes. Its efficacy has been demonstrated in tests with Drosophila melanogaster, particularly when the mutation rate between the reference and target species is minimal.

**BRAKER** is an evolving eukaryotic genome gene prediction pipeline. BRAKER1 (Hoff *et al.* 2016) combined GeneMark-ET and AUGUSTUS, using genome assemblies and RNA-Seq reads for unsupervised annotation. BRAKER2 (Brůna *et al.* 2021) added integration with a protein database, automating training for new genomes and surpassing MAKER in accuracy. The latest version, BRAKER3 (Gabriel *et al.* 2023, Gabriel *et al.* 2024), integrates both RNA-Seq and protein data, leveraging GeneMark-ETP, AUGUSTUS, and the TSEBRA combiner, setting a new standard in genome annotation precision.

**CEGMA** (Core Eukaryotic Genes Mapping Approach) (Parra *et al.* 2007) is a pipeline designed to create gene annotations without experimental data. It uses hidden Markov models to accurately identify exon–intron structures in new genomic sequences. While not as advanced as more recent pipelines, its simplicity and effectiveness made it a popular choice in the past.

**TGFam-Finder** (Kim *et al.* 2020), or Target Gene Family Finder, focuses on the structural annotation of plant genes encoding proteins with specific domains of interest. Alongside new gene models, it provides a platform for comprehensive functional, comparative, and evolutionary studies in plants.

**Seqping** (Chan *et al.* 2017) is a pipeline tailored for plant genome annotation. It integrates multiple tools and databases to deliver comprehensive gene annotations, including functions and pathways. Designed for usability, it is tailored for plants and offers a user-friendly interface, making it accessible to researchers without extensive bioinformatics expertise.

**ASPic-GeneID** (Alioto *et al.* 2013) combines the ASPic algorithm, which predicts alternative splicing isoforms, with the GeneId gene prediction tool. This integration enables the pipeline to predict both constitutive and alternative splicing isoforms within genomic sequences.

## 10 Major annotation providers

In genomic annotation, several institutions stand out as primary sources of annotated genomic data. Their methodologies, while sometimes not fully disclosed, significantly influence genomic research.

### NCBI’s approach and GNOMON

The National Center for Biotechnology Information (NCBI) ranks among the top authorities in genomic research. Their gene prediction pipeline, GNOMON, plays a crucial role in their annotation system. GNOMON integrates evidence from multiple sources, including ab initio predictions, protein homology, and transcript data. Although the detailed workings of GNOMON are not fully disclosed, its results constitute a large portion of the gene annotations available in the GenBank database.

### EBI and the ENSEMBL pipeline

The European Bioinformatics Institute (EBI) is another leader in the field. Its ENSEMBL project provides a wide-ranging suite of annotated genomes. The ENSEMBL pipeline uses diverse tools and techniques, tailored to different organisms and data types. Like NCBI, EBI combines multiple sources of evidence to produce reliable gene predictions. However, the exact prioritization and interactions between tools within the pipeline remain proprietary.

### JGI’s annotation pipeline

The Joint Genome Institute (JGI) is particularly recognized for its work on plant, fungal, and microbial genomics. Although the specifics of its annotation methods are less widely disclosed than those of NCBI and EBI, JGI’s annotations are highly regarded in the scientific community, especially for non-model organisms. Their approach likely integrates ab initio predictions, homology-driven methods, and empirical data.

### Transparency and accessibility issues

Despite the invaluable resources these institutions provide, there is a growing call for greater transparency in their annotation processes. Making these methods publicly available and easily accessible would enhance trust, foster collaboration, and promote innovation. Allowing the broader community to evaluate and build upon these pipelines could help establish stronger standards in gene prediction and accelerate advances in the field.

## 11 Evaluating and benchmarking gene prediction

Eukaryotic gene prediction tools often face challenges due to the unique structure of eukaryotic genes, which are interrupted by introns. While manual curation is invaluable, it is not feasible for large-scale projects such as the Earth BioGenome Project (Lewin *et al.* 2018). The influx of genomic data highlights the need for automated gene prediction. There has been a clear shift from manual annotation methods to automated computational approaches designed for large genomic datasets. The effectiveness of these tools is best evaluated through comparative analysis, using metrics such as sensitivity and specificity at different levels.

**Nucleotide level**: Predicted values are compared with actual nucleotide values. Sensitivity (Sn) and specificity (Sp) (Burset and Guigo 1996) are calculated using true positives (TPs: number of coding nucleotides predicted as coding), true negatives (TNs: number of non-coding nucleotides predicted as non-coding), false positives (FPs: number of non-coding nucleotides predicted as coding), and false negatives (FNs: number of coding nucleotides predicted as non-coding) (Burset and Guigo 1996).


$$\begin{array}{l} \text{Sn }=\text{ TP}/\left(\text{TP }+\text{ FN}\right)\\ \text{Sp }=\text{ TP}/\left(\text{TP }+\text{ FP}\right) \end{array}$$

**Exon structure level**: Predicted exons are matched with actual exons. A predicted exon and an actual exon are matched if their start positions and end positions are aligned. Based on this matching, sensitivity and specificity metrics are defined to measure accuracy. Since the definition of predicted and actual exons matching is strict, the missing exons (ME) score and the wrong exons (WE) score are additional measures (Burset and Guigo 1996, Scalzitti *et al.* 2020). The ME score is the ratio of actual exons without overlap to predicted exons to the total number of actual exons. The WE is the ratio of predicted exons without overlap to actual exons to the total number of predicted exons.

**Protein and transcript levels**: At the protein level, accuracy is determined by comparing the protein from the actual gene with the predicted protein. At transcript level, it is assessed by comparing predicted transcripts with verified ones. The comparison is based on the percent sequence identity, defined as the number of identical amino acids compared to the number of amino acids in the verified sequence. The sequence identity metric is the average sequence identity between the predicted sequence and the verified benchmark sequences is computed.

Benchmarking gene prediction tools is vital for accuracy assessment. Given the large number of tools, each designed for specific tasks, direct comparisons are challenging. These tools have diverse output formats and input requirements, complicating benchmarking. It is essential to compare tools with similar objectives for meaningful results.

**G3PO** (Benchmark for Gene and Protein Prediction Programs) (Scalzitti *et al.* 2020) was developed to evaluate modern genome annotation tools. The benchmark dataset comprises real annotated genomes from various eukaryote species, with high-confidence gene annotations. Through comparative analysis, strengths and weaknesses of gene model-based prediction tools, including GlimmerHMM, Augustus, GenScan, Snap, and GeneID, were identified. Augustus and GenScan were top performers, with Augustus requiring the most computational resources. No tool excelled in predicting short exons, and the phylogenetic location of sequences affected accuracy. The study suggests leveraging artificial intelligence to improve gene predictions.

**BUSCO** (Benchmarking Universal Single-Copy Orthologs) (Simão *et al.* 2015, Seppey *et al.* 2019) evaluates the quality of genome assemblies, gene sets, and transcriptomes by checking for universally conserved single-copy genes, providing a reliability metric for genomic and transcriptomic datasets.

**OMArk** (Nevers *et al.* 2025) is a more recent tool designed to evaluate the quality of protein-coding gene sets. Available as both a command-line and online tool, OMArk uses k-mer-based methods to classify proteins into gene families and subfamilies. From these classifications, it estimates species composition and assesses proteome consistency, thereby offering insights into genome annotation quality.

## 12 Experimental comparison of gene prediction tools

**Figure 2.**
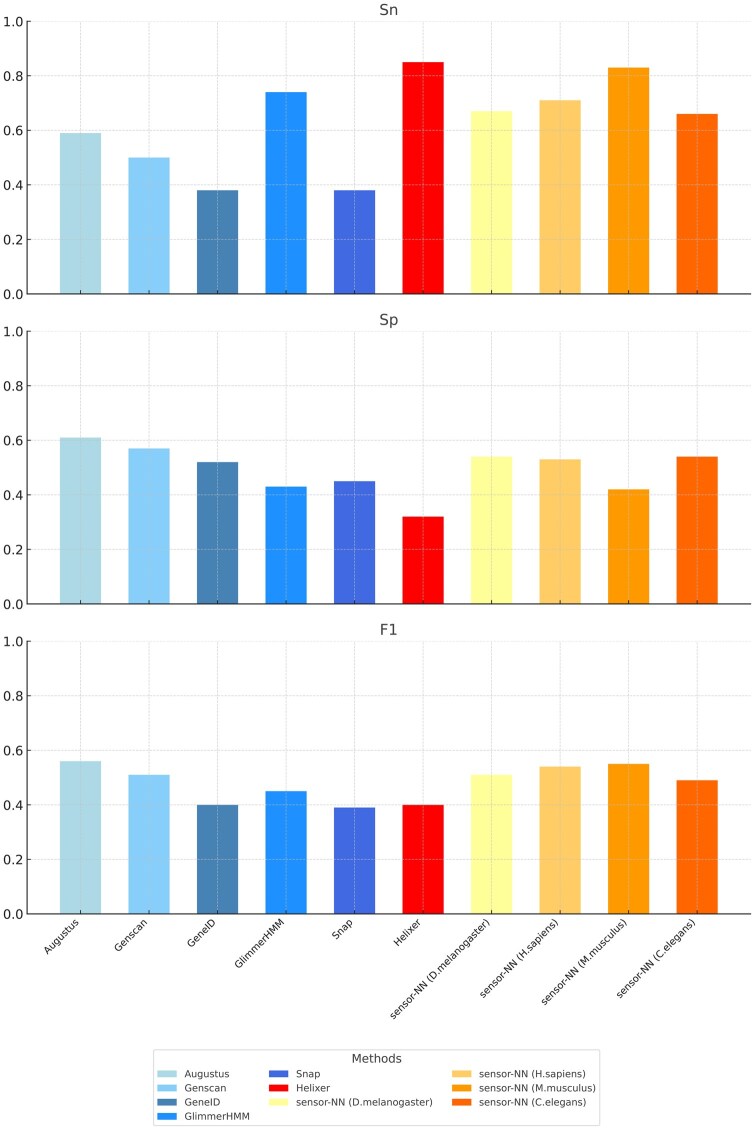
Nucleotide level performance for different methods.

**Figure 3.**
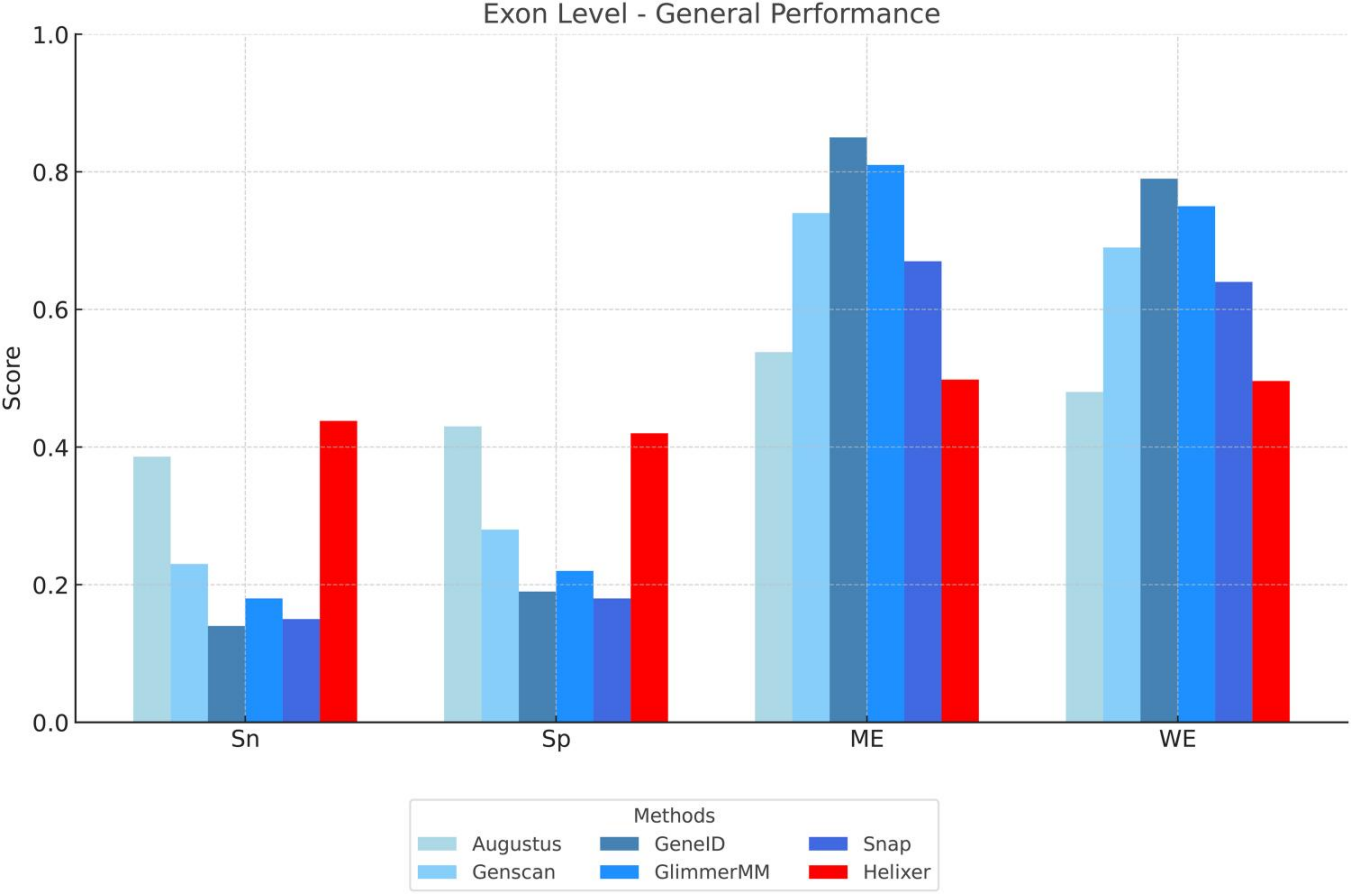
Exon level performance for different methods.

We extended the G3PO benchmark analysis (Scalzitti *et al.* 2020) on gene model-based prediction tools (Augustus, GenScan, GeneID, GlimmerHMM, and Snap) to include a gene model-free method, sensor-NN (Baker *et al.* 2023), and a hybrid method, Helixer (Holst *et al.* 2023). Figures 2 and 3 illustrate the performance of the tools at nucleotide and exon levels, respectively.

The results of GenScan, GeneID, GlimmerHMM, and Snap are from the initial G3PO benchmark (Scalzitti *et al.* 2020). For Augustus, the predictions were recalculated using a more recent version (v3.3.3). For sensor-NN, we included four versions of the method trained on four model species (D. melanogaster, H. sapiens, M. musculus, and C. elegans) with parameters set to maximize the F1-score.

### 12.1 Nucleotide level performance

At the nucleotide level, three metrics—Sensitivity (Sn), Specificity (Sp), and F1 score—are used for the comparison:

**Sensitivity (Sn):** Helixer, the hybrid method, achieves the highest sensitivity (0.85), demonstrating its effectiveness in correctly predicting true coding nucleotides. Sensor-NN (M. musculus F1), a gene model-free method, follows closely with a sensitivity of 0.83, while GlimmerHMM (0.74) and the other sensor-NN methods (0.66 to 0.71) also perform well. The other gene model-based methods show lower sensitivity, with Augustus scoring moderately at 0.59. In contrast, SNAP (0.38) and GeneID (0.38) exhibit the lowest sensitivity, indicating a high rate of missed coding nucleotides.

**Specificity (Sp):** Augustus achieves the highest specificity (0.61), closely followed by Genscan (0.57). Both perform well in correctly identifying non-coding nucleotides. The sensor-NN methods show moderate specificity scores, ranging from 0.42 (M. musculus F1) to 0.54 (D. melanogaster F1). GeneID (0.52) also demonstrates moderate specificity, while GlimmerHMM (0.43) and sensor-NN (M. musculus F1) (0.42) show lower specificity. In contrast to its high sensitivity, Helixer has the lowest specificity (0.32), indicating a higher rate of false positives.

**F1 Score:** All methods have a low to moderate F1 score. Augustus tops the F1 score (0.59), indicating the best balanced performance between sensitivity and specificity. Genscan (0.51) and the sensor-NN methods (0.49 to 0.55) have moderate scores. Helixer (0.46), and the other gene model-based predictors [GlimmerHMM (0.45), Snap (0.39), and GeneID (0.40)] have lower F1 scores, reflecting challenges in balancing sensitivity and specificity.

Helixer and the sensor-NN methods are good at predicting true coding nucleotides, as shown by their high sensitivity scores. This is likely due to their use of deep neural networks, which can capture complex patterns in gene structures, and their base-wise predictions, which help detect even weak or short signals—leading to fewer missed coding regions. In contrast, they perform less well at correctly identifying non-coding regions, resulting in more false positives and reduced specificity. This may be due to overfitting of the neural network models to the training data in the case of sensor-NN, or the weakness of the post-processing step—relying on biological structure and constraints to build full gene models after classifying nucleotides as coding or non-coding—in the case of Helixer. The low specificity of gene model-free methods is the reason they need to be extended into hybrid methods with structured post-processing to maintain good specificity.

Gene model-based methods, on the other hand, are typically less sensitive, mainly because of their simplified models of gene structure and their prioritization of biologically plausible gene models. This approach helps reduce false positives, improving specificity, but it can also lead to missing true coding regions, thus lowering sensitivity.

The contrasting performance of methods in terms of sensitivity and specificity is why hybrid methods and combiners are popular. The latter are designed to strike a balance between accurately identifying true coding regions and minimizing false positives.

### 12.2 Exon level performance

At the exon level, sensor-NN was excluded from the comparison because it does not produce complete gene structures. The focus of the sensor-NN method is on the accuracy of nucleotide-level classification, and the method is not yet fully developed to assemble whole genes. At the exon level, the comparison is based on four metrics: Sensitivity (Sn), Specificity (Sp), Missed Exons (ME), and Wrong Exons (WE).

**Sensitivity (Sn):** Helixer (0.44) shows the highest sensitivity, making it the most effective at correctly identifying exons. Augustus (0.39) also performs well, while Genscan (0.23) and GlimmerHMM (0.18) have moderate sensitivity. Snap (0.15) and GeneID (0.14) show lower sensitivity, indicating that they miss many true exons.

**Specificity (Sp):** Augustus (0.43) and Helixer (0.42) lead in specificity, effectively showing the false positives are minimized. Genscan (0.28) and GlimmerHMM (0.22) show moderate specificity, while Snap (0.18) and GeneID (0.19) have lower specificity, showing higher false positive rates.

**Missed Exons (ME):** Helixer (0.50) and Augustus (0.54) have fewer missed exons. Snap (0.67) and Genscan (0.74) miss more exons, while GlimmerHMM (0.81) and GeneID (0.85) have the highest numbers of missed exons.

**Wrong Exons (WE):** Augustus (0.48) and Helixer (0.50) have the lowest rates of wrong exons. Snap (0.64) and Genscan (0.69) have moderate levels of wrong exons, while GlimmerHMM (0.75) and GeneID (0.79) show the highest rates of incorrect exon predictions.

Overall, Helixer and Augustus stand out with the highest sensitivity and specificity, and the lowest ME and WE. Like at the nucleotide level, the results observed at the exon level suggest a trend where the hybrid approach (Helixer) achieves superior sensitivity compared to traditional gene model-based approaches. In terms of sensitivity, Helixer remains the best at identifying true exon sequences, as shown by its highest sensitivity score and lowest ME score. In contrast to Helixer’s poor specificity performance at the level of nucleotides, Helixer is now the second best after Augustus at the level of exons, compared to the other gene model-based methods, which perform significantly less well in their specificity score and WE score.

Regarding the sensitivity performance of gene model-based approaches, the latter might be constrained by the limitations of their predefined models, which can hinder their ability to detect all true positives, leading to lower sensitivity and higher rates of ME. However, as represented by Augustus, they can have higher specificity and lower WE as they rely on well-established biological principles encoded in their models.

These results highlight the evolving landscape of gene prediction tools, where integrating hybrid techniques can enhance the sensitivity and overall performance of gene prediction.

## 13 Discussion and conclusion

In the last two decades, computational gene prediction has advanced considerably. Although many reviews categorize gene prediction tools according to the type of information they use (intrinsic or extrinsic), this review primarily adopts a categorization according to their methodology. It distinguishes between gene model-based approaches, gene model-free methods, and hybrid strategies. Gene model-based methods rely on predefined gene structure models, whereas model-free methods learn the gene model from the data. Hybrid methods combine elements of both gene model-based and gene model-free approaches. The review also discusses the case of combiners and pipelines that integrate multiple tools to enhance gene prediction accuracy.

Several traditional gene model-based tools are still widely used today. Augustus (Stanke *et al.* 2004, 2006a,b, 2008, Stanke and Morgenstern 2005, Hoff and Stanke 2013), GeneWise/GenomeWise (Birney *et al.* 2004), Genscan (Burge and Karlin 1997), SNAP (Korf 2004), GlimmerHMM and TigrScan (Majoros *et al.* 2004) are among the most commonly used and highly cited tools (see Table 6, available as supplementary data at *Bioinformatics Advances* online for their number of citations since 2015). The most widely used category of tools today, however, consists of combiners and pipelines, including BUSCO (Simão *et al.* 2015, Seppey *et al.* 2019, Waterhouse *et al.* 2018), CEGMA (Parra *et al.* 2007), EvidenceModeler (Haas *et al.* 2008) and Maker (Cantarel *et al.* 2008, Holt and Yandell 2011). These tools integrate the strengths of multiple predictors to improve the accuracy of gene predictions, though often at the cost of increased computational time.

In practice, even the best gene prediction tools leave room for improvement in the accuracy of eukaryotic gene prediction. Tools like Augustus or GeneMark-ES can identify about 70%–85% of protein-coding genes in well-studied organisms such as human and Arabidopsis, but only around 20%–30% of those predictions are full-length correct gene models, including UTRs and accurate exon-intron junctions. Most gene predictions contain small errors, often in the UTR regions or at splice sites. Evidence-based methods generally achieve higher accuracy than ab initio methods. For instance, Augustus with RNA-seq hints can identify about 85%–95% of human protein-coding genes, with 25%–40% full-length correctly predicted gene models (Brůna *et al.* 2021, Gabriel *et al.* 2021). Combiner tools like EVidenceModeler, JIGSAW, or GLEAN often provide more accurate predictions than individual methods alone. They produce more precise and complete gene annotations by balancing the weaknesses of each individual method. When run with high-quality evidence data, combiner tools can identify up to 98% of human protein-coding genes, with 60% full-length predicted gene models (Holt and Yandell 2011, Zickmann and Renard 2015).

Previous works have shown that gene model-based methods, especially Augustus, tend to produce fewer false positives, especially in well-studied genomes, because of their use of well-established gene structure models (Scalzitti *et al.* 2020). However, they often miss atypical gene signals, and they are especially poor at detecting short exons and non-canonical splice sites. Combining multiple tools and diverse data sources generally improves prediction accuracy (Keilwagen *et al.* 2019, Gabriel *et al.* 2021). As genomic data becomes more accessible, gene model-free approaches are gaining attention, offering more accurate gene prediction with increased sensitivity. However, the accuracy of their predictions still largely depends on the quality and quantity of the data provided (Mathé *et al.* 2002).

Despite the development of numerous tools and significant improvements in addressing gene prediction challenges, some well-documented issues remain unresolved or only partially addressed.

First, several prediction tools rely on discriminative statistical characteristics, which often leads to missed short exons. Gao and Zhang (2004) emphasized the effectiveness of the Z-curve method for such exons. Yet, Saeys *et al.* (2007) suggested that merging complementary sequence features can surpass conventional models like the Z-curve, particularly for short sequences. Mabrouk (2012) showed the potential of frequency domain techniques, such as the EIIP mapping method, in exon prediction. Despite these advancements, short exon prediction remains challenging. Gene model-free prediction approaches might offer enhanced solutions as more data on short exons become available. In this context, Zhang *et al.* (2016) introduced a gene model-free method leveraging singularity detection with wavelet transform modulus maxima for short exon detection in eukaryotic DNA sequences, showing promising results. Detecting short exons is crucial, given their significant role in protein function and essential biological processes (Zhang and Pan 2019).

Second, gene prediction often faces issues due to incomplete gene models leading to mis-predictions, particularly when overlooking aspects such as alternative splicing and non-canonical splice sites (Meyer and Durbin 2002). Most tools primarily predict a single transcript for each gene, not fully capturing transcript diversity. In this context, incomplete gene models mean missing transcript isoforms rather than truncated genomic genes. Some tools handle this but predict transcripts individually. A promising strategy is to integrate alternative/constitutive splice sites into gene model formulations. For instance, Hidden Markov Models (HMMs) could incorporate alternative splicing states, enabling prediction of multiple isoforms within a single framework (Fawal *et al.* 2014).

Third, the metrics used to evaluate gene prediction tools have been consistently highlighted as a limitation in prior reviews. The common practice of comparing prediction method accuracy involves sensitivity and specificity across nucleotides, exons, and proteins. However, there is a need to refine evaluation measures and develop combined scores that integrate these aspects, while reflecting biological processes like alternative splicing.

Fourth, the creation of error-free benchmark datasets, representing the diversity of gene models and organisms, is vital. The G3PO benchmark (Scalzitti *et al.* 2020) was established to compare programs across 147 eukaryotic genomes, but it excludes alternative splicing and targets mainly ab initio tools. More refined benchmarks are needed, possibly using simulated data to capture broader diversity.

Fifth, standalone tools often exhibit limitations. Integrating multiple predictors and data sources consistently improves outcomes (Allen *et al.* 2004). Hybrid approaches show particular promise, with methods like HelixerInit (model-free) and HMM-based post-processing representing current trends. Majoros and Salzberg (2007) emphasized machine learning classification views of gene prediction, which may dominate future approaches.

The introduction of newer prediction techniques, particularly model-free approaches, has come with challenges. These methods rely heavily on selecting meaningful features and training data quality (Claverie 1997). Studies on feature representations, such as Scalzitti *et al.* (2020) and Zielezinski *et al.* (2017), highlight the need for innovation beyond k-mers.

The abundance of sequence data since the Human Genome Project (Wang *et al.* 2004) provides opportunities but also quality issues (Sleator 2010, Goel *et al.* 2013). Databases like GenBank and Ensembl attempt to filter errors (Pennisi 1999, Mathé and Dunand 2021), but manual oversight remains essential. Tools like BUSCO (Waterhouse *et al.* 2018), OMArk (Nevers *et al.* 2025), and recent studies (Kwon *et al.* 2023) stress the importance of high-quality training sets for accuracy.

Emerging deep learning architectures (RNNs, Attention, Transformers) offer transformative possibilities. RNNs capture sequential dependencies but face gradient issues; Attention enables dynamic focus on sequence regions; Transformers efficiently model long-range dependencies but require high resources. Their integration into pipelines must balance accuracy and computational feasibility.

Lastly, it must be recognized that all gene prediction methods are developed based on the existing understanding of gene expression and molecular biology (Wang *et al.* 2004). As new discoveries emerge, they provide additional information that should be incorporated into prediction tools to boost their accuracy.

## Supplementary Material

vbaf222_Supplementary_Data

## Data Availability

The DNA and protein sequences used in the benchmark are available in the *G3PO repository* at http://git.lbgi.fr/scalzitti/Benchmark\_study. The scripts used to produce the results for Augustus (König *et al.* 2016) and Helixer (Holst *et al.* 2023), and the outputs of the prediction are available at https://github.com/UdeS-CoBIUS/GenePredictionReviewBenchmark.
