# Supplementary material for: An overview of computational methods for gene prediction in eukaryotes: strengths, limitations, and future directions
Source: Bioinform Adv. 2025 Oct 1;5(1):vbaf222. doi: 10.1093/bioadv/vbaf222 (PMC12701808; doi:10.1093/bioadv/vbaf222)
Supplement: vbaf222_Supplementary_Data [file vbaf222_supplementary_data.docx]

# Supplementary Material

**Table 1. Model-based gene prediction programs.**

This table summarizes programs that rely on predefined probabilistic models such as HMMs and GHMMs. Each entry specifies the approach used, the type of information exploited (intrinsic, extrinsic, or combined), and whether the program supports prediction of multiple transcripts.

**Table 2. Model-free gene prediction programs.**

This table lists methods that do not depend on explicit probabilistic models, often using neural networks, discriminative training, or decision trees. The type of information used and transcript support are indicated.

**Table 3. Hybrid gene prediction programs.**

This table includes programs that combine model-based and model-free approaches, leveraging both intrinsic genomic signals and extrinsic evidence such as homology or alignments.

**Table 4. Combiners and pipelines.**

This table presents integrative frameworks that combine multiple prediction programs or organize them into pipelines. Each entry specifies the tools included and the type of integration (combiner, pipeline, or comparative tool).

**Table 5. Original organisms for training data.**

This table lists the organisms used for the initial training of gene prediction programs. Asterisks indicate programs that have been retrained on other organisms. For each organism, the associated program and the database used are provided.

**Table 6. Availability of gene prediction programs (as of June 20, 2025).**

This table reports the accessibility and licensing of the programs, including whether a web server, software package, or source code is available. The license type and the number of citations since 2015 are also indicated.

## Table 1. Model-based Gene Prediction Programs

| Programs | Approaches used | Type of information | Multiple transcripts |
| --- | --- | --- | --- |
| Augustus (Stanke et al., 2003) | GHMM | Intrinsic | No |
| Augustus+ (Stanke et al., 2006, 2008) | GHMM | Intrinsic + Extrinsic | Yes |
| Augustus-PPX (Keller et al., 2011) | GHMM | Intrinsic + Extrinsic | Yes |
| Augustus_CGP (König et al., 2016) | GHMM | Intrinsic + Extrinsic | Yes |
| CESAR (Sharma et al., 2016) | HMM | Intrinsic | No |
| CESAR 2.0 (Sharma et al., 2017, 2019) | HMM | Intrinsic + Extrinsic | No |
| Conrad (Decaprio et al., 2007) | Semi-Markov conditional random fields (SMCRFs) | Intrinsic + Extrinsic | No |
| CRAIG (Bernal et al., 2007) | HMM + Conditional random field model | Intrinsic | No |
| DOUBLESCAN (Meyer et al., 2002) | HMM | Intrinsic + Extrinsic | Yes |
| EuGène (Schiex et al., 2000) | Weighted Directed Acyclic Graph + IMM | Intrinsic | No |
| EuGène'HOM (Foissac et al., 2003) | Weight Array Model + 2nd order Markov model | Intrinsic + Extrinsic | No |
| EuGène-M (Foissac et al., 2005) | Graph + DP + Alignment based method | Intrinsic + Extrinsic | Yes |
| Exonerate (Slater et al., 2005) | Bounded Sparse Dynamic Programming (BSDP) | Intrinsic + Extrinsic | No |
| ExonHunter (Brejova et al., 2005) | HMM | Intrinsic + Extrinsic | Yes |
| Fgene, Fgeneh (Solovyev et al., 1995) | Dynamic Programming + Linear Discriminant | Intrinsic | No |
| Fgenesh (Salamov et al., 2000) | HMM | Intrinsic | No |
| Fgenesh+ (Salamov et al., 2000) | HMM | Intrinsic + Extrinsic | No |
| GAP III (Xu et al., 1994) | Dynamic Programming | Intrinsic | No |
| GAZE (Howe et al., 2002) | GHMM + Posterior probabilities | Intrinsic + Extrinsic | No |
| GeMoMa (Keilwagen et al., 2016, 2019) | Alignments + Dynamic Programming | Extrinsic | No |
| GeneBuilder (Milanesi et al., 1999) | Dynamic Programming | Intrinsic + Extrinsic | Yes |
| GeneID (Guigo et al., 1992) | Dynamic Programming | Intrinsic | No |
| GeneMark (Borodovsky et al., 1993) | Non-homogeneous Markov chain model | Intrinsic | No |
| GeneMark-ES (Lomsadze et al., 2005) | HMM | Intrinsic | No |
| GeneMark-ET (Lomsadze et al., 2014) | HMM | Extrinsic | No |
| GeneMark.hmm (Lukashin et al., 1998) | HMM | Intrinsic | No |
| GeneMarkETP (Brůna et al., 2024) | HMM | Extrinsic | Yes |
| GeneScout (Yin et al., 2004) | HMM + Directed Acyclic Graph | Intrinsic | No |
| GenomeThreader (Gremme et al., 2005) | Dynamic Programming + Intron Cutout Technique | Intrinsic + Extrinsic | Yes |
| GenomeScan (Yeh et al., 2001) | HMM | Intrinsic + Extrinsic | Alternative exons |
| GenomeWise (Birney et al., 2004) | HMM + Dynamic Programming | Intrinsic + Extrinsic | No |
| GenLang (Dong et al., 1994) | Grammar Rule | Intrinsic | No |
| Genviewer (Milanesi et al., 1993) | Dynamic Programming + Maximum coding potential | Intrinsic | No |
| GenScan (Burge et al., 1997) | HMM | Intrinsic | No |
| GeneWise (Birney et al., 2004) | HMM + Dynamic Programming | Intrinsic + Extrinsic | No |
| GlimmerHMM (Majoros et al., 2004) | HMM | Intrinsic | No |
| GlimmerM (Salzberg et al., 1999) | Dynamic Programming + Interpolated Markov Model | Intrinsic | No |
| GREAT (Gelfand et al., 1996) | Dynamic Programming + Scoring function | Intrinsic | No |
| HMMgene (Krogh et al., 1997) | HMM + Conditional Maximum Likelihood | Intrinsic | No |
| JIGSAW (Allen et al., 2005) | Dynamic Programming | Intrinsic + Extrinsic | No |
| Miniprot (Li et al., 2023) | K-mers + Dynamic Programming | Intrinsic + Extrinsic | No |
| N-Scan (Gross et al., 2006) | HMM + Phylogeny | Intrinsic + Extrinsic | No |
| Phat (Cawley et al., 2001) | HMM | Intrinsic | No |
| ROSETTA (Batzoglou et al., 2000) | Cross-species sequence comparison | Extrinsic | No |
| Scipio (Keller et al., 2008) | Alignments | Extrinsic | No |
| SGP-1 (Wiehe et al., 2001) | Alignments | Extrinsic | No |
| SLAM (Cawley et al., 2003) | HMM | Intrinsic | Yes |
| SNAP (Korf et al., 2004) | HMM | Intrinsic | No |
| TigrScan (Majoros et al., 2004) | HMM | Intrinsic | No |
| Twinscan (Korf et al., 2001) | HMM + Alignment based approach | Intrinsic + Extrinsic | No |

## Table 2. Model-free Gene Prediction Programs

| Programs | Approaches used | Type of information | Multiple transcripts |
| --- | --- | --- | --- |
| AGenDA (Rinner et al., 2002; Taher et al., 2003; Taher et al., 2004) | Long-range alignments | Extrinsic | No |
| AnABlast (Jimenez et al., 2015; Rubio et al., 2019; Casimiro et al., 2020) | Alignments | Extrinsic | No |
| CODEX (Roberts et al., 1995) | Artificial Neural Network | Extrinsic | No |
| eCRAIG (Bernal et al., 2012) | Discriminative training | Intrinsic + Extrinsic | No |
| genBlastDT (She et al., 2010) | Decision Trees | Extrinsic | No |
| GRAIL1 (Uberbacher et al., 1991) | Artificial Neural Network | Extrinsic | No |
| RescueNet (Mahony et al., 2004) | Self-Organizing Map | Extrinsic | No |
| HelixerInit (Stiehler et al., 2020) | Convolutional layers + bLSTM layers | Intrinsic | No |
| sensor-NN (Baker et al., 2023) | Neural network modeling + features extraction | Intrinsic | No |

## Table 3. Hybrid Gene Prediction Programs

| Programs | Approaches used | Type of information | Multiple transcripts |
| --- | --- | --- | --- |
| CONTRAST (Gross et al., 2007) | Multiple Alignments | Extrinsic | No |
| GeneParser (Snyder et al., 1995) | Dynamic Programming + Neural networks | Intrinsic + Extrinsic | No |
| Genie (Reese et al., 2000) | HMM + Neural Networks | Intrinsic | No |
| GIN (Cai et al., 1998) | Artificial Neural Network | Extrinsic | No |
| GRAIL2 (Shah et al., 1994) | Artificial Neural Network | Extrinsic | No |
| Helixer (Holst et al., 2023) | Convolutional layers + bLSTM layers + HMM | Intrinsic | No |
| mGene (Schweikert et al., 2009) | HMM + SVM | Intrinsic | No |
| MORGAN (Salzberg et al., 1998) | Markov chains + DP + Decision Trees | Intrinsic | No |
| Tiberius (Gabriel et al., 2024) | CNN + LSTM + HMM | Intrinsic | No |

## Table 4. Combiners and Pipelines

| Programs | Programs included | Type |
| --- | --- | --- |
| ASPic-GeneID (Alioto et al., 2013) | GeneID and ASPic | Pipeline |
| BRAKER1 (Hoff et al., 2016) | GeneMark-ET and Augustus | Pipeline |
| BRAKER2 (Brůna et al., 2021) | GeneMark-EP+ and Augustus | Pipeline |
| BRAKER3 (Gabriel et al., 2024) | GeneMark-ETP, Augustus and TSEBRA | Pipeline |
| BUSCO (Simao et al., 2015; Seppey et al., 2019) | HMMER and Augustus | Pipeline |
| CEGMA (Parra et al., 2007) | HMMER, GeneWise, and GeneId | Pipeline |
| Combiner (Allen et al., 2004) | GlimmerM, GeneMark.hmm, Genscan, GeneSplicer and TwinScan | Combiner |
| EVidenceModeler (Haas et al., 2008) | Fgenesh, GlimmerHMM, and GeneMark.hmm | Combiner |
| Evigan (Liu et al., 2008) | Augustus, GeneMarkk, Genscan, and SNAP | Combiner |
| GAF (Keilwagen et al., 2018) | GeMoMa | Combiner |
| GALBA (Brůna et al., 2023) | Miniprot and Augustus | Pipeline |
| GeneScope (1998) | FEXH, GeneParser, GENSCAN, and GRAIL2 | Combiner |
| GENFOCS (Anto et al., 2008) | Genscan, GlimmerHMM, GeneID, Glimmer2 and GeneMark.hmm | Comparative tool |
| GLEAN (Elsik et al., 2007) | Fgenesh, Ensembl, NCBI, Evolutionary Conserved Core, and Drosophila Ortholog Set | Combiner |
| MAKER (Cantarel et al., 2008; Holt et al., 2011) | SNAP, Augustus, and GeneMark | Combiner and Pipeline |
| Seqping (Chan et al., 2017) | GlimmerHMM, SNAP, Augustus, and MAKER2 | Pipeline |
| TGFam-Finder (Kim et al., 2020) | Exonerate and Augustus | Pipeline |
| TSEBRA (Gabriel et al., 2021) | BRAKER1 and BRAKER2 | Combiner |

## Table 5. Original Organisms for training data

| Organism | Program | Database |
| --- | --- | --- |
| Animal | Helixer (Stiehler et al., 2020; Holst et al., 2023) | EnsemblMetazoa |
| Arabidopsis | GenomeScan (Yeh et al., 2001) | Nonredundant Protein database (GenPept + PDB + SwissProt + PIR) |
|  | GenomeThreader (Gremme et al., 2005) | NCBI, EMBL |
|  | GenScan (Burge et al., 1997) | GenBank (Burset/Guigó set of 570 vertebrate multi-exon gene sequences) |
| C. elegans | GeneMark.hmm (Lukashin et al., 1998) | GenBank |
|  | AnABlast (Jimenez et al., 2015) | WormBase (1 February 2014) |
|  | GeneBuilder (Milanesi et al., 1999) | SWISSPROT, ALLSEQ (Burset & Guigó, 1996) |
|  | mGene (Schweikert et al., 2009) | NCBI Nucleotide database |
|  | *SNAP (Korf et al., 2004) | TIGR, Ensembl |
| C. neoformans | Conrad (Decaprio et al., 2007) | GenBank |
| Drosophila | *Augustus (Stanke et al., 2003) | GenBank |
|  | *Augustus_CGP (König et al., 2016) | Flybase |
|  | Fgene (Solovyev et al., 1995) | NCBI NR protein + BDGP EST |
|  | GeneBuilder (Milanesi et al., 1999) | SWISSPROT, ALLSEQ (Burset & Guigó, 1996) |
|  | Genie (Reese et al., 2000) | GenBank |
| Human | *Augustus; Augustus+ (Stanke et al., 2003; 2006; 2008) | GenBank, dbEST, NCBI |
|  | *Augustus-PPX (Keller et al., 2011) | DHC Data |
|  | CESAR (Sharma et al., 2016) | UCSC |
|  | CODEX (Roberts et al., 1995) | GenBank |
|  | DOUBLESCAN (Meyer et al., 2002) | Jareborg et al., 1999 data |
|  | ExonHunter (Brejova et al., 2005) | 117 human single-gene sequences (Batzoglou et al., 2000) |
|  | GeneParser (Snyder et al., 1995) | GenBank + EST database |
|  | GenomeScan (Yeh et al., 2001) | GenBank, SingleGene dataset (Guigó, 2000) |
|  | *GeneMark (Borodovsky et al., 1993) | GenBank |
|  | GeneWise (Birney et al., 2004) | SWISS-PROT/SPTREMBL |
|  | GRAIL1 (Uberbacher et al., 1991) | GenBank |
|  | GREAT (Gelfand et al., 1996) | NCBI, Ensembl |
|  | HMMGene (Krogh et al., 1997) | GenBank |
|  | JIGSAW (Allen et al., 2005) | UCSC, Swiss-Prot, UniGene, TIGR Gene Index |
|  | Projector (Meyer et al., 2004) | RefSeq (10 Feb 2003) |
|  | ROSETTA (Batzoglou et al., 2000) | Own data |
|  | SLAM (Cawley et al., 2003) | UCSC |
|  | Twinscan (Korf et al., 2001) | GenBank |
| Maize | *GeneMark (Borodovsky et al., 1993) | GenBank |
|  | GenScan (Burge et al., 1997) | GenBank (Burset/Guigó set of 570 vertebrate multi-exon gene sequences) |
| Mouse | *Augustus-PPX (Keller et al., 2011) | DHC Data |
|  | CODEX (Roberts et al., 1995) | GenBank |
|  | DOUBLESCAN (Meyer et al., 2002) | Jareborg et al., 1999 |
|  | GeneBuilder (Milanesi et al., 1999) | SWISSPROT, ALLSEQ (Burset & Guigó, 1996) |
|  | *GeneMark (Borodovsky et al., 1993) | GenBank |
|  | GenomeScan (Yeh et al., 2001) | GenBank, SingleGene (Guigó, 2000) |
|  | GRAIL1 (Uberbacher et al., 1991) | GenBank |
|  | GREAT (Gelfand et al., 1996) | NCBI, Ensembl |
|  | Projector (Meyer et al., 2004) | RefSeq (10 Feb 2003) |
|  | ROSETTA (Batzoglou et al., 2000) | Own data |
|  | SLAM (Cawley et al., 2003) | UCSC |
|  | Twinscan (Korf et al., 2001) | GenBank |
| Plants | CODEX (Roberts et al., 1995) | GenBank |
|  | GeneID (Guigo et al., 1992) | GenBank |
|  | GlimmerHMM (Majoros et al., 2004) | TAIR, Gramene |
|  | Helixer (Stiehler et al., 2020; Holst et al., 2023) | Phytozome |
|  | SGP-1 (Wiehe et al., 2001) | GenBank |
|  | SplicePredictor | GenBank |
| Plasmodium falciparum | GlimmerM (Salzberg et al., 1999) | PlasmoDB |
|  | Phat (Cawley et al., 2001) | Sanger Centre; WEHI |
| Vertebrates | *Augustus_CGP (König et al., 2016) | UCSC Genome Browser database |
|  | CESAR (Sharma et al., 2016) | UCSC |
|  | GeneBuilder (Milanesi et al., 1999) | SWISSPROT, ALLSEQ (Burset & Guigó, 1996) |
|  | GeneID (Guigo et al., 1992) | GenBank |
|  | GeneParser (Snyder et al., 1995) | GenBank + EST database |
|  | GeneScout (Yin et al., 2004) | GenBank |
|  | GenScan (Burge et al., 1997) | GenBank (Burset/Guigó set of 570 vertebrate multi-exon gene sequences) |
|  | GIN (Cai et al., 1998) | GenBank |
|  | HMMgene (Krogh et al., 1997) | GenBank |
|  | MORGAN (Salzberg et al., 1998) | Burset and Guigó database (1998) |
|  | SGP-1 (Wiehe et al., 2001) | GenBank |
| Worm | GAZE (Howe et al., 2002) | WormBase WS52 (September 2001) |

## Table 6. Availability (As of June 20th, 2025)

| Programs (last update) | Web Server? | Software available? | Source code available? | License | Citations since 2015 |
| --- | --- | --- | --- | --- | --- |
| AGenDA (Rinner et al., 2002) | No | No | No | - | 3 |
| AGenDA (Taher et al., 2003) | No | No | No | - | 16 |
| AGenDA (Taher et al., 2004) | No | No | No | - | 2 |
| AnABlast (Jimenez et al., 2015) | Yes | No | No | - | 16 |
| AnABlast (Rubio et al., 2019) | Yes | No | No | - | 7 |
| ASPic-GeneID (Alioto et al., 2013) | No | No | No | - | 13 |
| Augustus (Stanke et al., 2003) (2023) | Yes | Yes | Yes - C++ | GPL | 1390 |
| Augustus+ (Stanke et al., 2006) | Yes | Yes | Yes - C++ | GPL | 2400 |
| Augustus-PPX (Keller et al., 2011) | Yes | Yes | Yes - C++ | GPL | 582 |
| Augustus_CGP (König et al., 2016) | Yes | Yes | Yes - C++ | GPL | 73 |
| BRAKER1 (Hoff et al., 2016) | No | Yes | Yes | - | 1140 |
| BRAKER2 (Brůna et al., 2021) | No | Yes | Yes | - | 1536 |
| BRAKER3 (Gabriel et al., 2024) | No | Yes | Yes | - | 314 |
| BUSCO1 (Simao et al., 2015) | No | Yes | Yes - Python | MIT | 12360 |
| BUSCO2 (Seppey et al., 2019) | No | Yes | Yes - Python | MIT | 2022 |
| CEGMA (Parra et al., 2007) | No | Yes | Yes | GPL | 2060 |
| CESAR (Sharma et al., 2016) (2017) | No | Yes | Yes - Python | MIT | 54 |
| CESAR 2.0 (Sharma et al., 2017, 2019) (2022) | No | Yes | Yes - C | MIT | 46 |
| Combiner (Allen et al., 2004) | No | No | No | - | 46 |
| Conrad (Decaprio et al., 2007) | No | No | No | GPL | 37 |
| CONTRAST (Gross et al., 2007) (2007) | No | Yes | Yes - C and C++ | Open source | 48 |
| CRAIG (Bernal et al., 2007) (2007) | No | No | No - Perl | - | 43 |
| DOUBLESCAN (Meyer et al., 2002) | No | No | No | - | 22 |
| eCRAIG (Bernal et al., 2012) | No | No | No | - | 9 |
| EuGène (Schiex et al., 2000) | Yes | No | No - Java | - | 23 |
| EuGène'HOM (Foissac et al., 2003) | No | No | No - Perl | - | 15 |
| ExonHunter (Brejova et al., 2005) | No | No | No | - | 7 |
| EVidenceModeler (Haas et al., 2008) | No | Yes | Yes | BSD-3-Clause | 3200 |
| Evigan (Liu et al., 2008) | No | Yes (binary) | No | - | 31 |
| Fgene (Solovyev et al., 1995) (2016) | Yes | Yes (Not free) | No | Softberry License | 14 |
| Fgenesh (Salamov et al., 2000) | Yes | Yes (Not free) | No | Softberry License | 512 |
| GAF (Keilwagen et al., 2018) | No | No | No | - | 299 |
| GALBA (Brůna et al., 2023) | No | Yes | Yes | GPL-3.0 | 52 |
| GAP III (Xu et al., 1994) | No | No | No | - | 2 |
| GAZE (Howe et al., 2002) (2017) | No | No | No - C | - | 32 |
| GeMoMa (Keilwagen et al., 2016) (2022) | No | Yes | Yes - Java | GPL-3.0 | 620 |
| GeMoMa (Keilwagen et al., 2019) (2022) | No | Yes | Yes - Java | GPL-3.0 | 372 |
| genBlastDT (She et al., 2010) | No | No | No | - | 3 |
| GeneBuilder (Milanesi et al., 1999) | No | No | No | - | 12 |
| GeneID (Guigo et al., 1992) (2023) | Yes | Yes | Yes - C | GPL-3.0 | 89 |
| GeneMark (Borodovsky et al., 1993) | Yes | Yes | No (registration) | GeneMark Family | 222 |
| GeneMark.hmm (Lukashin et al., 1998) (2018) | Yes | Yes | Yes - C++ | GeneMark Family | 649 |
| GeneMark-EP (Brůna et al., 2020) | No | Yes | No (registration) | GeneMark Family | 430 |
| GeneMark-ES (Lomsadze et al., 2005) | No | Yes | No (registration) | GeneMark Family | 797 |
| GeneMark-ET (Lomsadze et al., 2014) | No | Yes | No (registration) | GeneMark Family | 563 |
| GeneMark-ETP (Brůna et al., 2023) | No | Yes | No (registration) | GeneMark Family | 34 |
| GeneParser (Snyder et al., 1995) | No | No | No - C | - | 29 |
| GeneScope (1998) | No | No | No | - | 8 |
| GeneScout (Yin et al., 2004) | No | No | No | - | 7 |
| GeneWise (Birney et al., 2004) (2022) | Yes | Yes | Yes - C and Perl | - | 1850 |
| Genie (Reese et al., 2000) | No | No | No | - | 29 |
| GENFOCS (Anto et al., 2008) | No | No | No | - | 0 |
| GenLang (Dong et al., 1994) | No | No | No | - | 27 |
| GenomeScan (Yeh et al., 2001) (2002) | Yes | No | No | - | 100 |
| GenomeThreader (Gremme et al., 2005) (2020) | No | Yes | Yes - C | Not licensed | 296 |
| GenomeWise (Birney et al., 2004) | No | No (Ensembl) | No | - | 1850 |
| GenScan (Burge et al., 1997) (2009) | Yes | No | Yes (registration) | Academic use | 1500 |
| Genviewer (Milanesi et al., 1993) | No | No | No | - | 3 |
| GIN (Cai et al., 1998) | No | No | No | - | 4 |
| GLEAN (Elsik et al., 2007) | No | No | No | - | 219 |
| GlimmerHMM (Majoros et al., 2004) | No | Yes | Yes - C | Artistic | 1550 |
| GlimmerM (Salzberg et al., 1999) | No | No | No | - | 44 |
| GRAIL (Gelfand et al., 1996) | No | No | No | - | 1 |
| HelixerInit (Stiehler et al., 2020) (2023) | No | Yes | Yes - Python | GPL-3.0 | 80 |
| Helixer (Holst et al., 2023) (2023) | No | Yes | Yes - Python | GPL-3.0 | 47 |
| HMMgene (Krogh et al., 1997) | Yes | Yes (Fill a form) | No | Academic software license | 71 |
| JIGSAW (Allen et al., 2005) (2020) | No | No | No - C++ | Artistic-1.0 | 64 |
| MAKER (Cantarel et al., 2008) | No | Yes | Yes | GPL | 2086 |
| mGene (Schweikert et al., 2009) (2015) | No | Yes | Yes - C++ and MATLAB | MIT | 52 |
| Miniprot (Li et al., 2023) (2023) | No | Yes | Yes - C | MIT | 257 |
| MORGAN (Salzberg et al., 1998) (2017) | No | No | No | - | 28 |
| N-Scan (Gross et al., 2006) (2011) | No | No | No - C and Perl | - | 33 |
| Phat (Cawley et al., 2001) (2000) | No | Yes | Yes - Perl and C | GPL-3.0 | 4 |
| ROSETTA (Batzoglou et al., 2000) | No | No | No | - | 140 |
| Scipio (Keller et al., 2008) (2013) | Yes | Yes | Yes - Perl and Ruby | GPL | 119 |
| Seqping (Chan et al., 2017) | No | No | No | - | 49 |
| sensor-NN (Baker et al., 2023) | No | Yes (via publication) | Yes – Python (GitHub) | MIT | 6 |
| SGP-1 (Wiehe et al., 2001) | No | No | No | - | 16 |
| SLAM (Cawley et al., 2003) (2017) | No | No | No | - | 20 |
| SNAP (Korf et al., 2004) (2022) | No | Yes | Yes - C | GPL | 2920 |
| TGFam-Finder (Kim et al., 2020) | No | Yes | Yes | MIT | 26 |
| Tiberius (Gabriel et al., 2024) | No | Yes | Yes – Python | GPL-3.0 | 5 |
| TigrScan (Now GeneZilla) (Majoros et al., 2004) (2003) | No | Yes | Yes - C++ | Artistic | 1550 |
| TSEBRA (Gabriel et al., 2021) | No | Yes | Yes | Artistic 2.0 | 254 |
| Twinscan (Korf et al., 2001) | No | No | No - C and Perl | - | 76 |

**References**

Alioto, T.S., Blanco, E., Parra, G. & Guigó, R. (2013). ASPic-GeneID: a pipeline for the identification and annotation of alternative splicing events. Bioinformatics, 29(9), 1216–1218.

Allen, J.E. & Salzberg, S.L. (2005). JIGSAW: integration of multiple sources of evidence for gene prediction. Bioinformatics, 21(18), 3596–3603.

Allen, J.E., Pertea, M. & Salzberg, S.L. (2004). Computational gene prediction using multiple sources of evidence. Genome Research, 14(1), 142–148.

Anto, R.J., et al. (2008). GENFOCS: a comparative gene prediction tool. Journal of Biosciences, 33(1), 45–52.

Baker, D., et al. (2023). sensor-NN: neural network–based prediction of gene structures. Nucleic Acids Research, 51(4), 2014–2027.

Batzoglou, S., et al. (2000). Human and mouse gene structure prediction using cross-species sequence comparison. Genome Research, 10(7), 950–958.

Bernal, A., Crammer, K. & Hatzigeorgiou, A.G. (2007). Global discriminative training for higher accuracy in gene prediction. Bioinformatics, 23(4), 529–536.

Bernal, A., et al. (2012). Automated discriminative training for gene prediction programs. Genome Biology, 13(4), R44.

Birney, E., Clamp, M. & Durbin, R. (2004). GeneWise and GenomeWise. Genome Research, 14(5), 988–995.

Borodovsky, M. & McIninch, J.D. (1993). GeneMark: parallel gene recognition for both DNA strands. Computers & Chemistry, 17(2), 123–133.

Brůna, T., et al. (2020). GeneMark-EP+: eukaryotic gene prediction with self-training in the space of proteins and spliced alignments. Nucleic Acids Research, 48(19), 11256–11272.

Brůna, T., et al. (2021). BRAKER2: automatic eukaryotic genome annotation with GeneMark-EP+ and AUGUSTUS supported by a protein database. Nucleic Acids Research, 49(2), 112–118.

Brůna, T., et al. (2023). GeneMark-ETP and GALBA: improved gene prediction pipelines. Genome Biology, 24(1), 55.

Brůna, T., et al. (2024). GeneMark-ETP: robust integration of transcript and protein data for gene prediction. Nucleic Acids Research, 52(6), 3121–3134.

Brejová, B., Brown, D.G. & Vinar, T. (2005). ExonHunter: a comprehensive approach to gene finding. Bioinformatics, 21(Suppl 1), i57–i65.

Burge, C. & Karlin, S. (1997). Prediction of complete gene structures in human genomic DNA. Journal of Molecular Biology, 268(1), 78–94.

Burset, M. & Guigó, R. (1996). Evaluation of gene structure prediction programs. Genomics, 34(3), 353–367.

Cai, Y. & Bork, P. (1998). Homology-based gene prediction. Trends in Genetics, 14(8), 352–353.

Cantarel, B.L., et al. (2008). MAKER: an easy-to-use annotation pipeline designed for emerging model organism genomes. Genome Research, 18(1), 188–196.

Casimiro-Soriguer, C.S., et al. (2020). Using AnABlast for the annotation of novel genes. BMC Genomics, 21, 514.

Cawley, S.L. & Pachter, L. (2003). HMM-based gene structure prediction. Bioinformatics, 19(Suppl 2), ii26–ii33.

Cawley, S.L., et al. (2001). Phat: a gene finding program based on hidden Markov models. Bioinformatics, 17(Suppl 1), S199–S206.

Chan, K.L., et al. (2017). Seqping: gene prediction pipeline for plant genomes. BMC Bioinformatics, 18(1), 387.

Decaprio, D., et al. (2007). Conrad: gene prediction using conditional random fields. Genome Research, 17(9), 1389–1399.

Elsik, C.G., et al. (2007). Creating a honey bee consensus gene set. Genome Biology, 8(1), R13.

Foissac, S. & Schiex, T. (2005). Integrating alternative splicing detection into gene prediction. BMC Bioinformatics, 6, 25.

Foissac, S., et al. (2003). EuGène’HOM: eukaryotic gene prediction using multiple sources of evidence. Genome Research, 13(8), 1889–1897.

Gelfand, M.S. & Roytberg, M.A. (1996). Recognition of genes in DNA sequence with errors. Nucleic Acids Research, 24(19), 3618–3627.

Gelfand, M.S., Mironov, A.A. & Pevzner, P.A. (1996). Gene recognition via spliced sequence alignment. Proceedings of the National Academy of Sciences USA, 93(17), 9061–9066.

Gremme, G., et al. (2005). Engineering a software framework for eukaryotic gene prediction. Genome Biology, 6(1), R7.

Gross, S.S. & Brent, M.R. (2006). Using multiple alignments for gene structure prediction. Bioinformatics, 22(5), 616–623.

Gross, S.S., et al. (2007). CONTRAST: a discriminative, phylogeny-free approach to multiple informant de novo gene prediction. Genome Biology, 8(12), R269.

Guigó, R., Knudsen, S., Drake, N. & Smith, T. (1992). Prediction of gene structure. Journal of Molecular Biology, 226(1), 141–157.

Haas, B.J., et al. (2008). Automated eukaryotic gene structure annotation using EVidenceModeler. Genome Biology, 9(1), R7.

Hoff, K.J., et al. (2016). BRAKER1: unsupervised RNA-seq–based genome annotation with GeneMark-ET and AUGUSTUS. Bioinformatics, 32(5), 767–769.

Holst, F., et al. (2023). Helixer: de novo prediction of eukaryotic gene structures using deep learning. Genome Biology, 24(1), 95.

Holt, C. & Yandell, M. (2011). MAKER2: an annotation pipeline and genome-database management tool. BMC Bioinformatics, 12, 491.

Howe, K.L., et al. (2002). GAZE: a framework for the integration of gene prediction data. Genome Research, 12(9), 1418–1427.

Jammali, S., et al. (2019). Gene prediction methods: advances and challenges. Briefings in Bioinformatics, 20(6), 2149–2162.

Jimenez, R., et al. (2015). AnABlast: detecting protein-coding potential in genomic sequences. Nucleic Acids Research, 43(20), e118.

Keller, O., Kollmar, M., Stanke, M. & Waack, S. (2008). Scipio: using protein sequences to determine the precise exon–intron structures of genes and their orthologs. BMC Bioinformatics, 9, 278.

Keller, O., Odronitz, F., Stanke, M. & Waack, S. (2011). A novel hybrid gene prediction method employing protein multiple sequence alignments. Bioinformatics, 27(6), 757–763.

Keilwagen, J., et al. (2016). Using intron position conservation for homology-based gene prediction. Nucleic Acids Research, 44(9), e89.

Keilwagen, J., Hartung, F. & Grau, J. (2019). GeMoMa: homology-based gene prediction utilizing intron position conservation and RNA-seq data. Methods in Molecular Biology, 1962, 161–177.

Keilwagen, J., et al. (2018). Combining evidence from multiple sources for gene prediction. Bioinformatics, 34(24), 4352–4359.

Kim, D., et al. (2020). TGFam-Finder: an integrative gene prediction tool for identifying gene families. BMC Bioinformatics, 21(1), 115.

König, S., Romoth, L.W., Gerischer, L. & Stanke, M. (2016). Simultaneous gene finding in multiple genomes. Bioinformatics, 32(22), 3388–3395.

Korf, I. (2004). Gene finding in novel genomes. BMC Bioinformatics, 5, 59.

Korf, I., Flicek, P., Duan, D. & Brent, M.R. (2001). Integrating genomic homology into gene structure prediction. Bioinformatics, 17(Suppl 1), S140–S148.

Krogh, A. (1997). Two methods for improving performance of an HMM and their application for gene finding. Proceedings of the Fifth International Conference on Intelligent Systems for Molecular Biology, 5, 179–186.

Li, H. (2023). Miniprot: fast alignment of protein sequences to genome assemblies. Bioinformatics, 39(1), btac806.

Liu, Y., et al. (2008). EVIGAN: a hidden variable model for integrating gene evidence. Bioinformatics, 24(5), 597–605.

Lomsadze, A., Burns, P.D. & Borodovsky, M. (2014). Integration of mapped RNA-Seq reads into automatic training of eukaryotic gene finding algorithm. Nucleic Acids Research, 42(15), e119.

Lomsadze, A., Ter-Hovhannisyan, V., Chernoff, Y.O. & Borodovsky, M. (2005). Gene identification in novel eukaryotic genomes by self-training algorithm. Nucleic Acids Research, 33(20), 6494–6506.

Lukashin, A.V. & Borodovsky, M. (1998). GeneMark.hmm: new solutions for gene finding. Nucleic Acids Research, 26(4), 1107–1115.

Mahony, S., et al. (2004). Gene prediction using self-organizing maps. Bioinformatics, 20(1), 117–125.

Majoros, W.H., Pertea, M. & Salzberg, S.L. (2005). TigrScan and GlimmerHMM: two open source ab initio eukaryotic gene-finders. Bioinformatics, 21(9), 1658–1659.

Meyer, I.M. & Durbin, R. (2002). Comparative ab initio prediction of gene structures using pair HMMs. Bioinformatics, 18(10), 1309–1318.

Meyer, I.M. & Durbin, R. (2004). Gene structure prediction using HMMs with pair hidden variables. Bioinformatics, 20(11), 1712–1723.

Meyer, M., et al. (2020). Improved gene prediction through integrated homology and transcript evidence. Nucleic Acids Research, 48(12), 7001–7012.

Milanesi, L., et al. (1993). GenViewer: a program for gene structure visualization. Computers & Chemistry, 17(2), 125–134.

Milanesi, L., et al. (1999). GeneBuilder: automatic annotation of eukaryotic genes. Bioinformatics, 15(6), 563–574.

Parra, G., Blanco, E. & Guigó, R. (2000). GeneID in Drosophila. Genome Research, 10(4), 511–515.

Parra, G., et al. (2007). CEGMA: a pipeline to accurately annotate core genes in eukaryotic genomes. Genome Research, 17(3), 1080–1086.

Reese, M.G., et al. (2000). Genie: gene finding with a hidden Markov model. Genome Research, 10(4), 529–538.

Rinner, O., et al. (2002). AGENDA: gene prediction by comparative sequence analysis. Bioinformatics, 18(4), 610–615.

Roberts, R.J., et al. (1995). Training set design for neural network gene prediction. Bioinformatics, 11(2), 123–130.

Rubio, A., et al. (2019). AnABlast for detecting novel genes in genomes. Nucleic Acids Research, 47(12), e68.

Salzberg, S.L., Delcher, A.L., Kasif, S. & White, O. (1998). Microbial gene identification using interpolated Markov models. Nucleic Acids Research, 26(2), 544–548.

Salzberg, S.L., et al. (1999). Interpolated Markov models for eukaryotic gene finding. Bioinformatics, 15(5), 364–374.

Schiex, T., Moisan, A. & Rouzé, P. (2000). EuGène: an integrative gene finder for eukaryotes. Journal of Computational Biology, 7(3–4), 277–295.

Schweikert, G., et al. (2009). mGene: accurate SVM-based gene finding with discriminative training. Genome Biology, 10(1), R13.

Sharma, A., et al. (2016). CESAR: a tool for comparative gene annotation. Bioinformatics, 32(11), 1658–1660.

Sharma, A., et al. (2017). CESAR 2.0: accurate coding exon prediction with comparative evidence. Bioinformatics, 33(19), 3266–3268.

Sharma, A., et al. (2019). Improved comparative gene annotation using CESAR 2.0. Nucleic Acids Research, 47(6), e42.

She, R., et al. (2010). genBlastA and genBlastG: algorithms for mining homologous sequences. BMC Bioinformatics, 11, 62.

Simão, F.A., Waterhouse, R.M., Ioannidis, P., Kriventseva, E.V. & Zdobnov, E.M. (2015). BUSCO: assessing genome assembly and annotation completeness with single-copy orthologs. Bioinformatics, 31(19), 3210–3212.

Slater, G.S.C. & Birney, E. (2005). Automated generation of heuristics for biological sequence comparison. BMC Bioinformatics, 6, 31.

Snyder, E.E. & Stormo, G.D. (1993). Identification of coding regions in genomic DNA sequences: an application of dynamic programming and neural networks. Nucleic Acids Research, 21(3), 607–613.

Snyder, E.E. & Stormo, G.D. (1995). GeneParser: improved recognition of protein coding regions. Bioinformatics, 11(3), 299–306.

Stanke, M. & Waack, S. (2003). Gene prediction with a hidden Markov model and a new intron submodel. Bioinformatics, 19(Suppl 2), ii215–ii225.

Stanke, M., et al. (2006). AUGUSTUS: ab initio prediction of alternative transcripts. Nucleic Acids Research, 34(Web Server issue), W435–W439.

Stanke, M., et al. (2006). AUGUSTUS: improved gene prediction with extrinsic information. Genome Research, 16(8), 979–985.

Stanke, M., et al. (2008). Using native and syntenically mapped cDNA alignments to improve de novo gene finding. Bioinformatics, 24(5), 637–644.

Stiehler, F., et al. (2020). HelixerInit: deep learning–based gene prediction. Bioinformatics, 36(18), 4458–4465.

Wiehe, T., Gebauer-Jung, S., Mitchell-Olds, T. & Guigó, R. (2001). SGP-1: prediction and validation of homologous genes based on synteny. Genome Research, 11(9), 1574–1583.
